# Putative anaerobic transformation pathway of microcystin-RR inferred from ^15^N labeling and multi-omics in an enriched shrimp pond sediment microbial community

**DOI:** 10.1371/journal.pone.0355950

**Published:** 2026-08-19

**Authors:** Chuanyin Liu, Jingjing Zhang, Rui Chen, Xiangdong Bi, Wei Dai, Wenjie Zhao, Dajuan Zhang, Qian Wang, Xinyu Wang

**Affiliations:** 1 Key Laboratory of Aquatic-Ecology and Aquaculture of Tianjin, Tianjin Agricultural University, Tianjin, China; 2 Institute of Crop Germplasm and Biotechnology, Tianjin Academy of Agricultural Sciences, Tianjin, China; Ministry of Fisheries, Animal Husbandry and Dairying, INDIA

## Abstract

The degradation mechanism of MC-RR by an anaerobic degrading microbial community (ADMC), enriched from shrimp pond sediment, was investigated using LC-MS/MS, metagenomic, and metatranscriptomic analyses. Three key degradation products of ^15^N-labeled MC-RR were tentatively identified: Adda-Glu-Mdha-Ala (m/z 618.3216), a deamination product (m/z 600.2965), and Glu-MeAsp-Ala-Arg-CO_2_ (m/z 466.2233). The pathway was inferred to involve hydrolytic ring-opening at Arg-Adda or Ala-Arg bonds, followed by deamination, decarboxylation, and stepwise degradation into short peptides and amino acids. Metagenomics revealed Citrobacter amalonaticus as the core dominant species and Shewanella as a low-abundance but transcriptionally active genus. Metatranscriptomic differential expression analysis (1,648 DEGs; 460 upregulated, 1,188 downregulated) showed significant upregulation of non-canonical peptidases including U32 family (YhbU, UbiU), M23 family (MepM), and S9 family serine peptidases, suggesting the involvement of a non-canonical, *mlr*-independent peptidase system in MC-RR transformation. Genes of the dissimilatory nitrate reduction to ammonium (DNRA) pathway (*narG–nirB–nrfA*) and nitric oxide reductase (*norB*) were concurrently upregulated, a transcriptional pattern consistent with DNRA-linked nitrogen turnover and NO detoxification during degradation, although the corresponding nitrogen fluxes were not directly measured. Among quorum sensing (QS) systems, the AI-2 system exhibited the most pronounced transcriptional response, with AHLs, DSF, and c-di-GMP genes also concurrently upregulated, suggesting a multi-signal transcriptional response during degradation of this complex substrate. These findings provide an important theoretical reference for revealing the mechanisms of anaerobic degradation of microcystins (MCs) by complex microbial communities in situ environments, while also offering scientific data to support the targeted development of efficient MCs-degrading microbial community or specific MCs-degrading enzymes.

## Introduction

The widespread eutrophication of freshwater ecosystems has driven the global proliferation of cyanobacterial blooms, with *Microcystis* being one of the most prevalent bloom-forming cyanobacteria worldwide [1]. Toxigenic strains of *Microcystis* produce microcystins (MCs), a class of cyclic heptapeptide hepatotoxins that have attracted considerable attention due to their exceptional chemical stability and potent biological toxicity [2,3]. The World Health Organization has classified MCs as Group 2B carcinogens [4] and established a safety threshold of no more than 1 μg/L for MCs in drinking water [5].

Due to their cyclic structure, MCs are chemically stable and not easily eliminated [6,7]. Photodegradation and biodegradation are considered the primary elimination pathways for MCs in natural environments [8,9]. However, MCs cannot be directly photolyzed by sunlight, their photodegradation occurs only in the presence of photosensitizers [10,11]. In contrast, MCs-degrading bacteria are ubiquitously distributed in natural water bodies [12–14]. Consequently, biodegradation mediated by these bacteria is widely recognized as a key natural attenuation process and is regarded as an economical and environmentally friendly strategy for MCs elimination in aquatic environments.

Specifically, sediments act as a major repository, accumulating the majority of dissolved and particulate MCs from the water column through physical adsorption and chemical sedimentation [15]. Umehara et al. [16] reported that approximately 50% of waterborne MCs are ultimately deposited in sediments. Once deposited, MCs do not undergo significant spontaneous migration, and their environmental fate depends predominantly on biodegradation [17,18]. For instance, Jin et al. [19] reported that a microbial community isolated from sediments of Dianchi Lake completely degraded MC-RR and MC-LR within 3 days at initial concentrations of 50 mg/L and 30 mg/L, respectively. Similarly, Yang et al. [20] isolated the bacterial community YFMCD4 from Lake Taihu sediments, which exhibited an MC-LR degradation rate of 0.5 μg/(mL·h). Notably, MCs can also be efficiently degraded under anaerobic conditions. Chen et al. [21] studied MCs biodegradation under anoxic and aerobic conditions using lake sediments as inocula and found similar degradation profiles under both conditions. Specifically, MC-LR was rapidly degraded to below the detection limit within 2 days and 3 days, following a 2-day and 3-day lag phase, respectively. Zhu et al. [22] showed that an anaerobic bacterial community from Lake Taihu sediment achieved approximately 80% degradation of 5 mg/L MC-LR within 6 days. This phenomenon is environmentally relevant, as changes in vertical dissolved oxygen (DO) gradients across the sediment–water interface drive the formation of hypoxic and anoxic microenvironments, with DO typically becoming depleted within the uppermost millimeters of the sediment [23,24]. These findings indicate that anaerobic biodegradation may play a more significant role in natural MCs removal within sediments than previously recognized.

However, current research on MCs biodegradation mechanisms has primarily focused on the *mlr* and *paa* gene cluster-mediated pathways under aerobic conditions [14,25,26]. In contrast, the mechanisms underlying anaerobic degradation remain relatively limited and poorly understood. Notably, *mlr* genes have not been detected in any anaerobic isolates nor in anaerobic biodegradation processes in natural sediments [21,27], providing strong evidence that anaerobic degradation pathways are genetically and enzymatically distinct from the well-characterized aerobic *mlr* pathway. This distinction is further supported by preliminary findings suggesting that the initial cleavage site in anaerobic MC-LR degradation by strain CJ5 may differ from that in aerobic pathways (e.g., cleavage at the Ala-Leu bond rather than the Adda-Arg bond) [28]. Consequently, the functional genes responsible for anaerobic MCs degradation, the composition of the degradation enzyme systems, and the molecular basis of synergistic community degradation under anoxic conditions remain outstanding scientific questions requiring elucidation [29].

With the advancement of multi-omics technologies, metagenomics and metatranscriptomics have become powerful tools for unraveling complex microbial metabolic networks [30,31]. In addition, stable isotope labeling techniques enable precise tracking of the metabolic fluxes of target elements, offering direct evidence for the elucidation of degradation pathways [32]. In this study, MC-RR was labeled with the ^15^N isotope, and LC-MS/MS was employed to investigate the intermediate products formed during the anaerobic degradation of MC-RR by anaerobic degrading microbial community (ADMC), enriched from shrimp pond sediment. Furthermore, metagenomic and metatranscriptomic analyses were conducted to reveal the expression regulatory networks of key functional genes involved in MC-RR degradation by ADMC. This work provides a theoretical foundation for understanding the mechanisms underlying anaerobic microcystin (MCs) degradation by complex microbial communities in situ, and supports the development of anaerobic microorganism-based bioremediation technologies for MCs.

## Materials and methods

### ADMC, *Microcystis* strain and reagents

The anaerobic degrading microbial community (ADMC) utilized in this study was previously enriched from shrimp culture pond sediment by our laboratory [33]. *Microcystis aeruginosa* FACHB-1178 was obtained from the Freshwater Algae Culture Collection at the Institute of Hydrobiology, Chinese Academy of Sciences. The MC-RR standard was also provided by the Institute of Hydrobiology, Chinese Academy of Sciences. Sodium nitrate (Na^15^NO_3_, purity ≥98.5%, ^15^N abundance 99%) was purchased from Aladdin Biochemical Technology Co., Ltd. (Shanghai, China).

### Preparation of ^15^N-Labeled MC-RR

*M. aeruginosa* strain was inoculated into sterile BG11 medium, in which NaNO_3_ was replaced with an equimolar amount of Na^15^NO_3_. Cultures were maintained at 25 °C under a light intensity of 36 μmol·m ^−2^·s ^−1^ with a 12 h light/12 h dark cycle. Cell density was monitored using a hemocytometer. Upon reaching a density of 5 × 10^8^ cells/mL, the culture was subcultured at a 1:1 (v/v) ratio of algal culture to fresh Na^15^NO_3_-supplemented BG11 medium. Qualification and quantification of MC-RR were performed using an Agilent 1260 Infinity HPLC system equipped with an Agilent HC-C18 column. The detection wavelength was set at 238 nm, and the mobile phase consisted of methanol and ultrapure water containing 0.01% trifluoroacetic acid (TFA) (60:40, v/v) at a flow rate of 1 mL/min.

Preparative purification of ^15^N MC-RR was conducted using an Agilent 1200 Infinity system fitted with a semi-preparative Eclipse XDB-C18 column; the specific preparation conditions are detailed in S1 Table. The purity and ^15^N labeling efficiency of the isolated compound were verified by liquid chromatography-mass spectrometry (LC-MS) using an Agilent 1290 II-6545 Q-TOF instrument operated in positive electrospray ionization (ESI+) mode. The mobile phase consisted of ultrapure water with 0.1% formic acid and acetonitrile (65:35, v/v) at a flow rate of 0.3 mL/min, with a fragmentor voltage of 220 V.

### Analysis of ^15^N MC-RR degradation products by ADMC

The ADMC was cultivated in Widdel medium [34] supplemented with ^15^N MC-RR at a final concentration of 1 μg/mL. To maintain anaerobic conditions, three Anaero Packs were placed inside an MGC-7L anaerobic container. The cultures were incubated at 28 °C with shaking at 150 rpm. Three biological replicates were established for each experimental group, alongside a sterile control group consisting of autoclaved Widdel medium spiked with ^15^N MC-RR. The residual concentration of ^15^N MC-RR in the culture system was monitored at 24 h intervals. LC-MS was employed to verify ^15^N labeling and to characterize the degradation products. The ion source was operated in positive electrospray ionization (ESI+) mode, with a full scan range of m/z 100–1200. This concentration, although higher than the levels typically encountered in ambient surface waters, was deliberately selected to ensure sufficient accumulation of transient degradation intermediates for reliable LC-MS detection and pathway reconstruction, in line with previous mechanistic studies of MC biodegradation [19,22].

### Nucleic acid extraction from ADMC and high-throughput sequencing

At 6.5 h after ^15^N MC-RR addition, bacteria were harvested by centrifugation at 10,000 rpm for 5 min at 4 °C. Total DNA and total RNA were extracted separately using the E.Z.N.A.® Bacterial DNA/RNA Kit (Omega Bio-tek). Samples were subsequently submitted to Novogene Co., Ltd. (Beijing, China) for metagenomic and metatranscriptomic sequencing. Both metagenomic and metatranscriptomic sequencing were performed on the Illumina NovaSeq 6000 platform using a paired-end 150 bp (PE150) sequencing strategy.

### Metagenomic and metatranscriptomic analyses

Raw metagenomic reads were quality-filtered using fastp (v0.23.1) to remove adapters and low-quality sequences. Metagenomic co-assembly was performed using MEGAHIT (v1.1.2). Contigs were binned using MetaBAT2 (v2.15) based on tetranucleotide frequency and abundance coverage to obtain metagenome-assembled genomes (MAGs). MAGs quality was assessed using CheckM for completeness and contamination, and high-quality bins (completeness > 90%, contamination < 10%) were selected. Genome-based taxonomic classification was performed using GTDB-Tk. Open reading frame (ORF) prediction on assembled contigs was conducted using Prokka (v1.14), and a non-redundant gene catalog was constructed using CD-HIT (identity 95%). Functional annotation was performed by aligning non-redundant genes against the NR, KEGG, eggNOG, QSP [35], and MEROPS databases using DIAMOND (v2.1.6) with an e-value threshold of <1e-5.

Metatranscriptomic raw reads were quality-filtered using fastp (v0.23.1). Clean reads were aligned to the non-redundant gene catalog constructed from the metagenome using RSEM (v1.2.28) to obtain read counts and FPKM values for each gene. Differentially expressed genes (DEGs) were identified using edgeR with thresholds of |log_2_FC| ≥ 0.585 and FDR < 0.05. Enrichment analyses against the GO and KEGG databases were performed using clusterProfiler. Data visualization was conducted using R.

### Multi-omics integrated analysis

Metatranscriptomic clean reads were aligned to the genomic sequences of individual MAGs to quantify the transcriptional read counts attributed to each MAG and their proportions among total DEGs, thereby resolving the contribution of individual strains to overall community transcriptional activity. These data were integrated with metagenomic species abundance data to compare the correspondence between community abundance and transcriptional contribution of each strain.

Based on LC-MS-identified degradation products, potential peptide bond cleavage sites within the cyclic heptapeptide structure of MC-RR were inferred. Concurrently, MEROPS database annotations were used to obtain family classifications and known substrate specificity information for significantly upregulated peptidases in the metatranscriptome, including cleavable peptide bond types, preferred amino acid residues, and recognition capabilities for D-amino acids or special structural moieties. The cleavage sites indicated by degradation products were matched with the substrate preferences of upregulated peptidases to infer the potential catalytic roles of individual peptidases in the MC-RR degradation pathway.

## Results

### ^15^N MC-RR labeling efficiency

**Fig 1:**
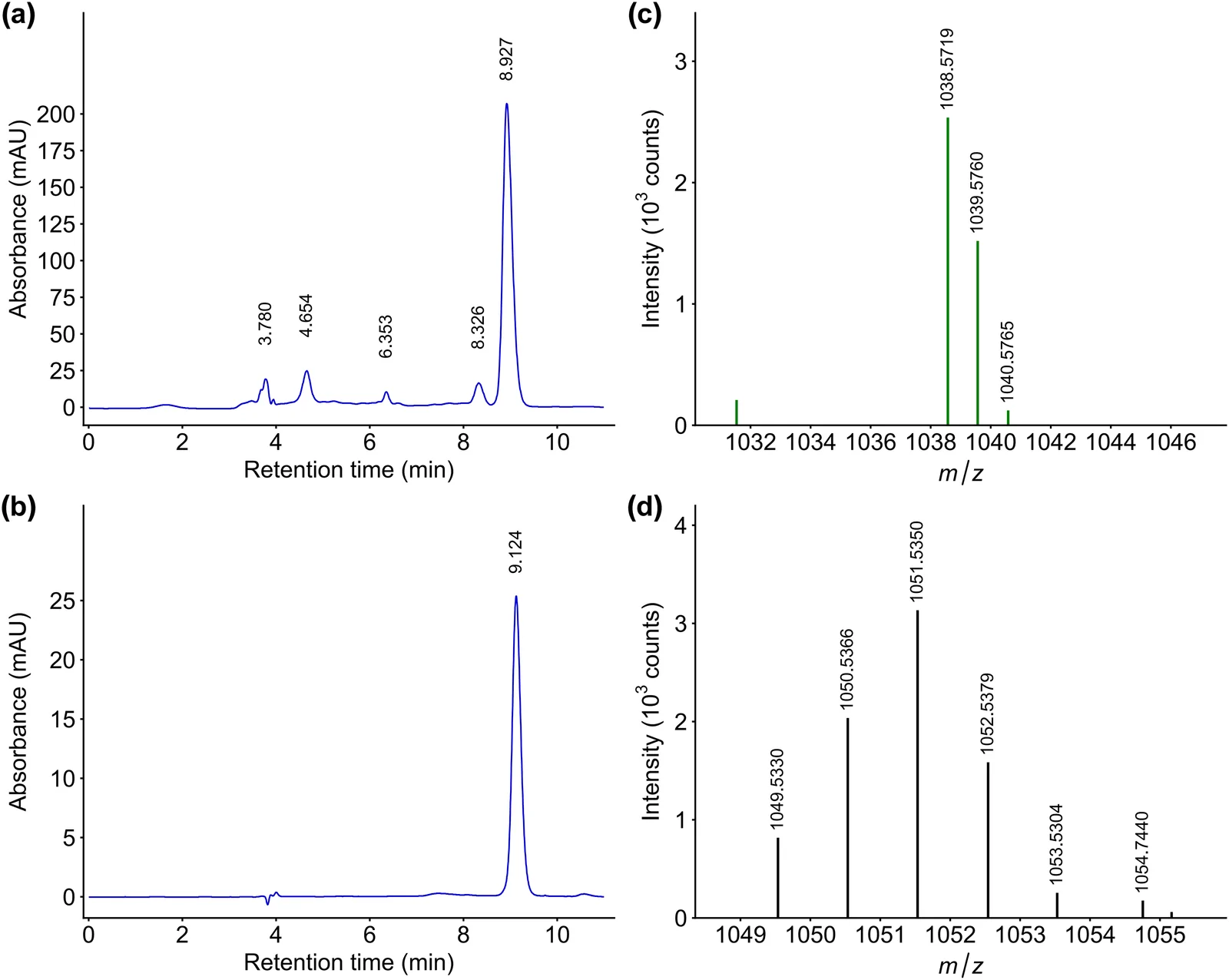
(a) HPLC chromatogram of crude ^15^N MC-RR extract, (b) HPLC chromatogram of preparative ^15^N MC-RR, (c) Mass spectrum of unlabeled MC-RR, (d) Mass spectrum of ^15^N-labeled MC-RR.

The MC-RR standard curve established by HPLC analysis is shown in S1 Fig. Using sonication combined with solid-phase extraction, 1026 μg MC-RR was extracted from 1 L of algal culture at a density of 5 × 10^8^ cells/mL (Fig 1a), and 915 μg MC-RR was ultimately recovered after preparative liquid chromatography (Fig 1b). LC-MS analysis of MC-RR revealed that the molecular ion peak [M + H]^+^ of unlabeled native MC-RR was 1038.5719 Da (Fig 1c), whereas the molecular ion peak of MC-RR after six generations of ^15^N cultivation shifted to 1051.5356 Da (Fig 1d). Given that the molecular formula of MC-RR is C_49_H_75_N_13_O_12_, containing 13 nitrogen atoms, the theoretical mass increase should be 13 × (15.0001–14.0031) ≈ 12.96 Da. The measured mass difference was 12.9637 Da, in close agreement with the theoretical value, confirming that all 13 nitrogen atoms in the MC-RR molecule had been replaced by ^15^N.

### Identification of ^15^N MC-RR degradation products

ADMC degraded MC-RR without a lag phase, initiating degradation immediately upon addition to the system, and MC-RR was degraded to below the detection limit within 72 h (S2 Fig). LC-MS (ESI + , MS1) analysis of samples at 0 h, 24 h, and 48 h identified 20 potential MC-RR degradation products. Based on the time-series peak area changes of 20 characteristic peaks across three time points: 11 features showed continuous increases (0 h < 24 h < 48 h),5 decreased after 24 h,3 were detected only at 24 h (m/z 466.2233, 303.2408, 600.2965), and 1 was detected only at 48 h (m/z 352.1166) (S2 Table).

Under constraints of CHNO elemental composition (C ≤ 49, ^15^N ≤ 13, O ≤ 14, H ≤ 90) and a mass error of ±5 ppm, 9 features yielded ^15^N molecular formula candidates: m/z 618.3216 → C_32_H_46_^15^N_4_O_8_; 855.6404 → C_45_H_85_^15^N_10_O_5_; 380.2312 → C_17_H_34_^15^N_2_O_7_; 279.1833 → C_12_H_26_^15^N_3_O_4_; 352.1166 → C_7_H_20_^15^N_8_O_8_; 466.2233 → C_18_H_33_^15^N_7_O_7_; 600.2965 → C_32_H_43_^15^N_3_O_8_; 603.3989 → C_27_H_58_^15^N_3_O_11_; 506.3468 → C_25_H_49_^15^N_3_O_7_. The remaining 11 masses (317.2927,229.2404,343.8684,457.8489,473.8270,241.1498,219.1472,303.2408,559.8136,673.7953,717.7693) had no matches within this elemental range.

By comparing the molecular formulas of detected MC-RR degradation products with their amino acid compositions, three key primary degradation products were identified. Product 1 (m/z 618.3216, molecular formula C_32_H_46_^15^N_4_O_8_) was identified as Adda-Glu-Mdha-Ala; Product 2 (m/z 600.2965, molecular formula C_32_H_43_^15^N_3_O_8_) represented Product 1 with loss of one ammonia molecule, likely a secondary degradation product formed through deamination of the linear tetrapeptide. Product 3 (m/z 466.2233, molecular formula C_18_H_33_^15^N_7_O_7_) was identified as Glu-MeAsp-Ala-Arg-CO_2_.

Based on MS mass matching results, ^15^N MC-RR was tentatively inferred to undergo hydrolytic ring-opening at the Arg-Adda or Ala-Arg peptide bond, generating an Adda-containing tetrapeptide and an arginine-rich tripeptide, followed by secondary modifications including deamination and dehydrogenation to form derivatives such as m/z 600.2965 and m/z 466.2233. These structural assignments were based on accurate-mass (MS1) matching within ±5 ppm and are therefore tentative, because the MS/MS fragmentation and NMR data required for unambiguous confirmation of the cleavage sites were not obtained in this study.

### Metagenomic characterization of ADMC community structure and functional features

**Fig 2:**
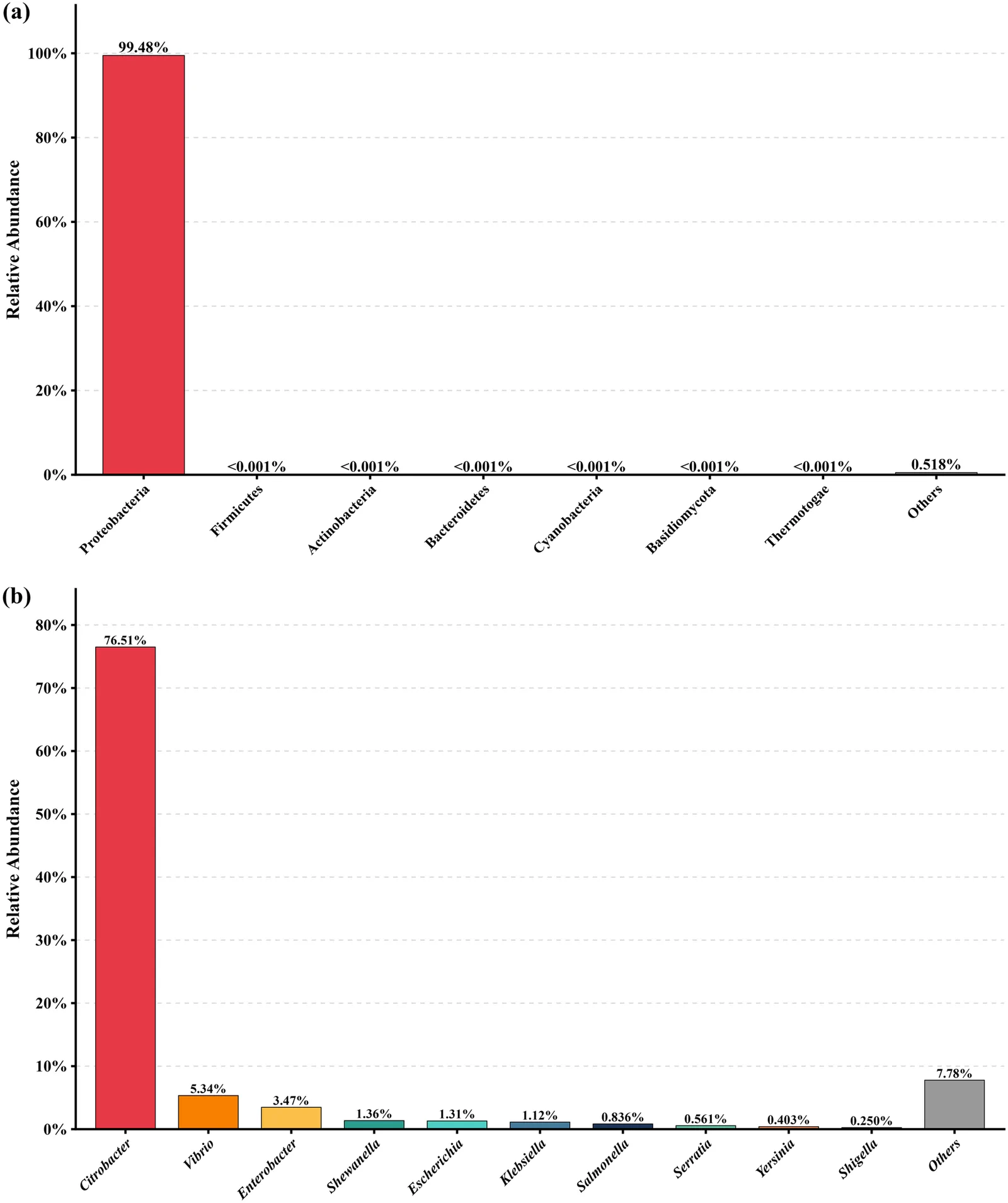
(a) Stacked bar chart of phylum-level relative abundance from ADMC metagenome (b) Stacked bar chart of genus-level relative abundance from ADMC metagenome.

Metagenomic sequencing generated 11.11 Gb of clean data with Q20 reaching 97.16% and GC content of 53.45%, providing a reliable foundation for subsequent bioinformatic analyses. Taxonomic annotation revealed that the anaerobic degrading community was overwhelmingly dominated by Proteobacteria at the phylum level, with a relative abundance of 99.48%, (Fig 2a). At the genus level, *Citrobacter* was the dominant genus with a relative abundance of 76.51%, followed by *Vibrio* (5.34%), *Enterobacter* (3.47%), and *Shewanella* (1.36%), indicating that the community formed a stable degradation consortium centered on *Citrobacter*, (Fig 2b).

**Fig 3:**
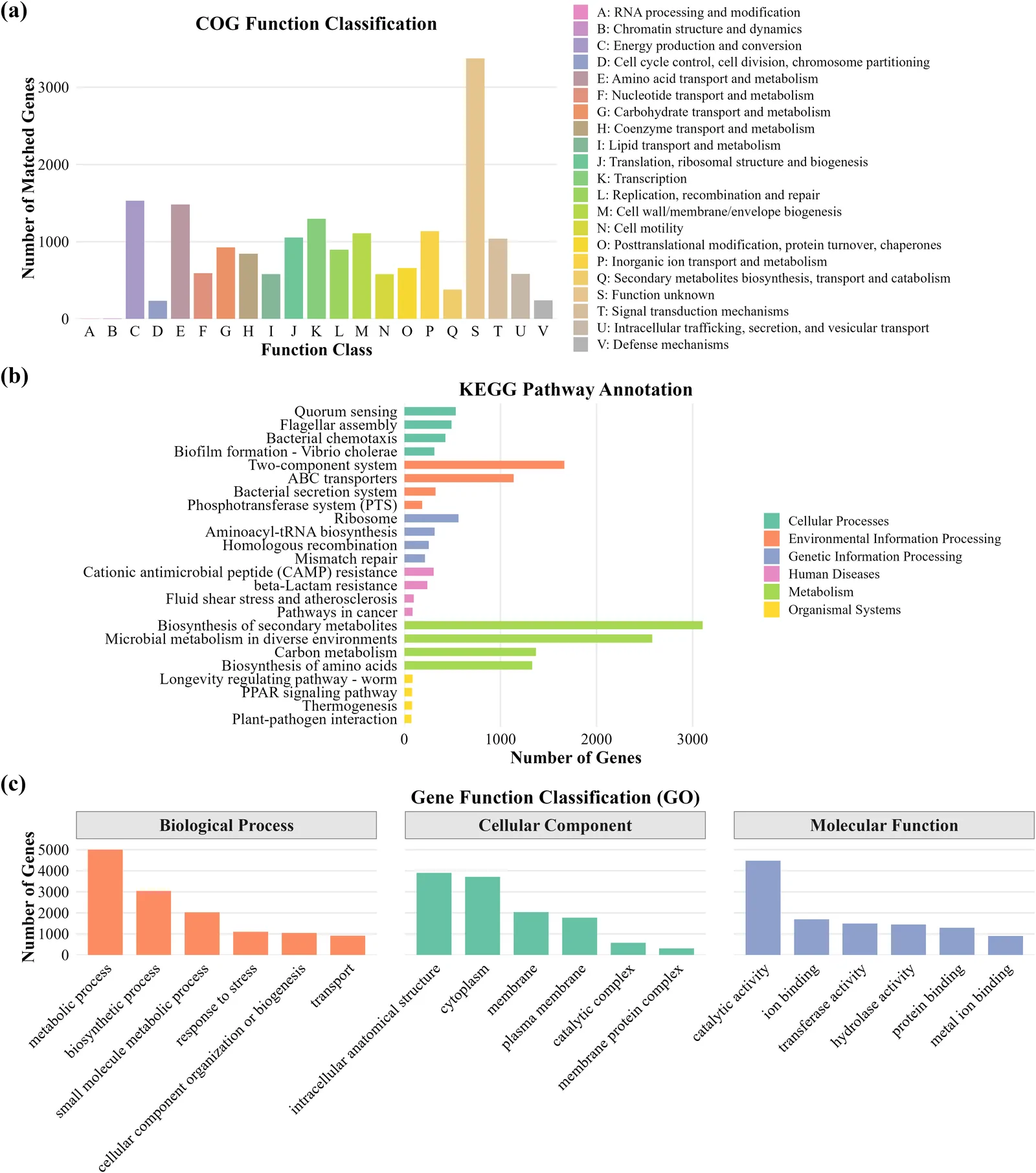
(a) Unigene eggNOG level 1 annotation results, (b) Unigene KEGG level 2 annotation results, (c) Unigene GO level 2 annotation results.

Functional annotation analysis revealed the complex metabolic potential of the community under anaerobic conditions. In COG functional classification, excluding the function unknown category (class S, 3375 genes, 18.7%), the most abundant gene categories were concentrated in metabolism and cellular processes. Energy production and conversion (class C,1530 genes), amino acid transport and metabolism (class E,1484 genes), and inorganic ion transport and metabolism (class P,1137 genes) were significantly enriched, indicating active material metabolism and energy conversion capabilities. KEGG pathway analysis further elucidated the metabolic network characteristics of the community. Metabolism-related pathways occupied a dominant position. In the global and overview maps, metabolic pathways (ko01100) and biosynthesis of secondary metabolites (ko01110) encompassed 6932 and 3104 genes, respectively, reflecting the robust biochemical synthesis and catabolic capacity of the community. In terms of environmental adaptability, signal transduction and membrane transport-related genes were significantly enriched, with two-component systems (ko02020,1664 genes) and ABC transporters (ko02010,1138 genes) present at high abundance, indicating that the community can sensitively perceive environmental chemical signals (e.g., toxins or nutrient changes) and efficiently transport substrates. Furthermore, the detection of xenobiotics biodegradation and metabolism-related genes, including benzoate degradation (ko00362), provided a potential genetic basis for MC-RR biodegradation. Collectively, this anaerobic community possesses complete functional modules at the genomic level for degrading complex organic pollutants, conserving energy, and responding to environmental stress, (Fig 3).

### Functional characterization of dominant strains based on binning analysis

Binning analysis of metagenomic data yielded five bins (Table 1), comprising two medium-quality and three high-quality MAGs: bin11 (*Serratia ureilytica*, completeness 67.50%, contamination 0.00%), bin15 (*Pseudomonas balearica*, completeness 51.07%, contamination 0.00%), bin5 (*Citrobacter amalonaticus*, completeness 99.97%, contamination 0.04%), bin12 (*Shewanella chilikensis*, completeness 100%, contamination 0.54%), and bin8 (*Shewanella algae*, completeness 90.7%, contamination 1.98%). The recovery of high-quality MAGs provided a reliable genomic basis for resolving the specific metabolic functions of community members.

**Table 1:** Summary statistics of metagenomic binning results.

bin Id	Completeness%	Contamination%	Taxonomy	Total length (Mbp)	Gene Numbers
bin12	100.00	0.54	*Shewanella chilikensis*	3.9	4141
bin5	99.97	0.04	*Citrobacter amalonaticus*	4.3	4667
bin8	90.70	1.98	*Shewanella algae*	4.4	5926
bin11	67.50	0.00	*Serratia ureilytica*	2.2	2578
bin15	51.07	0.00	*Pseudomonas balearica*	2.0	2973

Functional annotation of high-quality MAGs (S3–S5 Figs) revealed that the genome of *C. amalonaticus* (bin5) was significantly enriched in nutrient transport and basal metabolism. In COG classification, aside from function unknown genes (class S, 939 genes), carbohydrate transport and metabolism (class G,418 genes) and amino acid transport and metabolism (class E,383 genes) were the most abundant categories. KEGG pathway analysis further confirmed that bin5 harbored high-abundance ABC transporters (448 genes) and phosphotransferase system (PTS,148 genes), and was enriched in core carbon metabolism pathways including glycolysis/gluconeogenesis (134 genes) and pyruvate metabolism (116 genes). Additionally, this strain encoded enzyme systems related to benzoate degradation (62 genes) and drug metabolism (40 genes), indicating its metabolic potential for processing complex organic substrates.

In contrast, the two *Shewanella* strains exhibited distinctive genomic features in energy metabolism and environmental adaptability. The COG functional profile of *S. chilikensis* (bin12) showed high proportions of energy production and conversion (class C,314 genes) and cell wall/membrane biogenesis (class M,250 genes), and KEGG annotation revealed enrichment in multiple energy metabolism pathways including oxidative phosphorylation, sulfur metabolism, and nitrogen metabolism. *S. algae* (bin8) demonstrated strong environmental sensing capabilities, with COG signal transduction mechanisms (class T,332 genes) significantly more abundant than in other MAGs, and KEGG pathways containing numerous two-component system genes (440 genes) and bacterial chemotaxis/biofilm formation-related genes. These data suggest that *Shewanella* species may play critical roles in maintaining anaerobic community stability and adapting to environmental stress through efficient energy conversion systems and sensitive signal transduction networks.

### Metatranscriptomic differential expression analysis and identification of key functional genes

**Fig 4:**
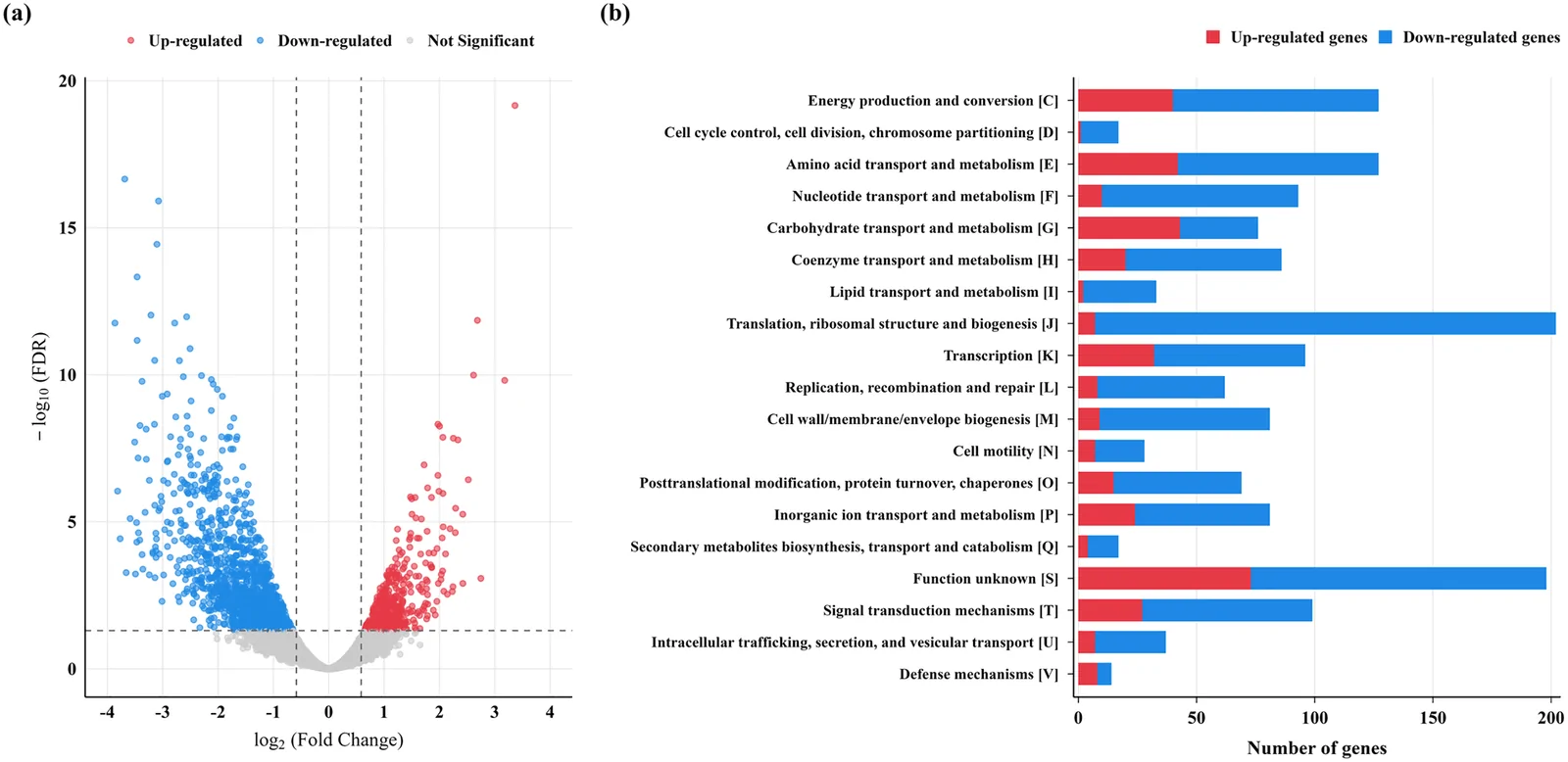
(a) Volcano plot of metatranscriptomic differentially expressed genes, (b) eggNOG classification of metatranscriptomic differentially expressed genes.

Metatranscriptomic sequencing yielded an average of 3.90 × 10^7^ raw reads and 11.70 Gb raw bases. After quality control, an average of 3.74 × 10^7^ clean reads and 11.22 Gb clean bases were obtained, with Q20 of 98.22%, Q30 of 94.95%, and GC content of 53.09%. Differential expression analysis identified a total of 1648 DEGs, of which 460 were significantly upregulated and 1188 were significantly downregulated (Fig 4a). In COG functional classification, upregulated genes were primarily concentrated in categories S (15.9%), G (9.3%), E (9.1%), C (8.7%), K (7.0%), T (5.9%), P (5.2%), and H (4.3%), suggesting overall enhancement of amino acid metabolism, energy conversion, transport, and regulation (Fig 4b). Strain-of-origin analysis revealed that upregulated genes were predominantly derived from *C. amalonaticus* (65%) and *S. chilikensis* (approximately 28%).

**Fig 5:**
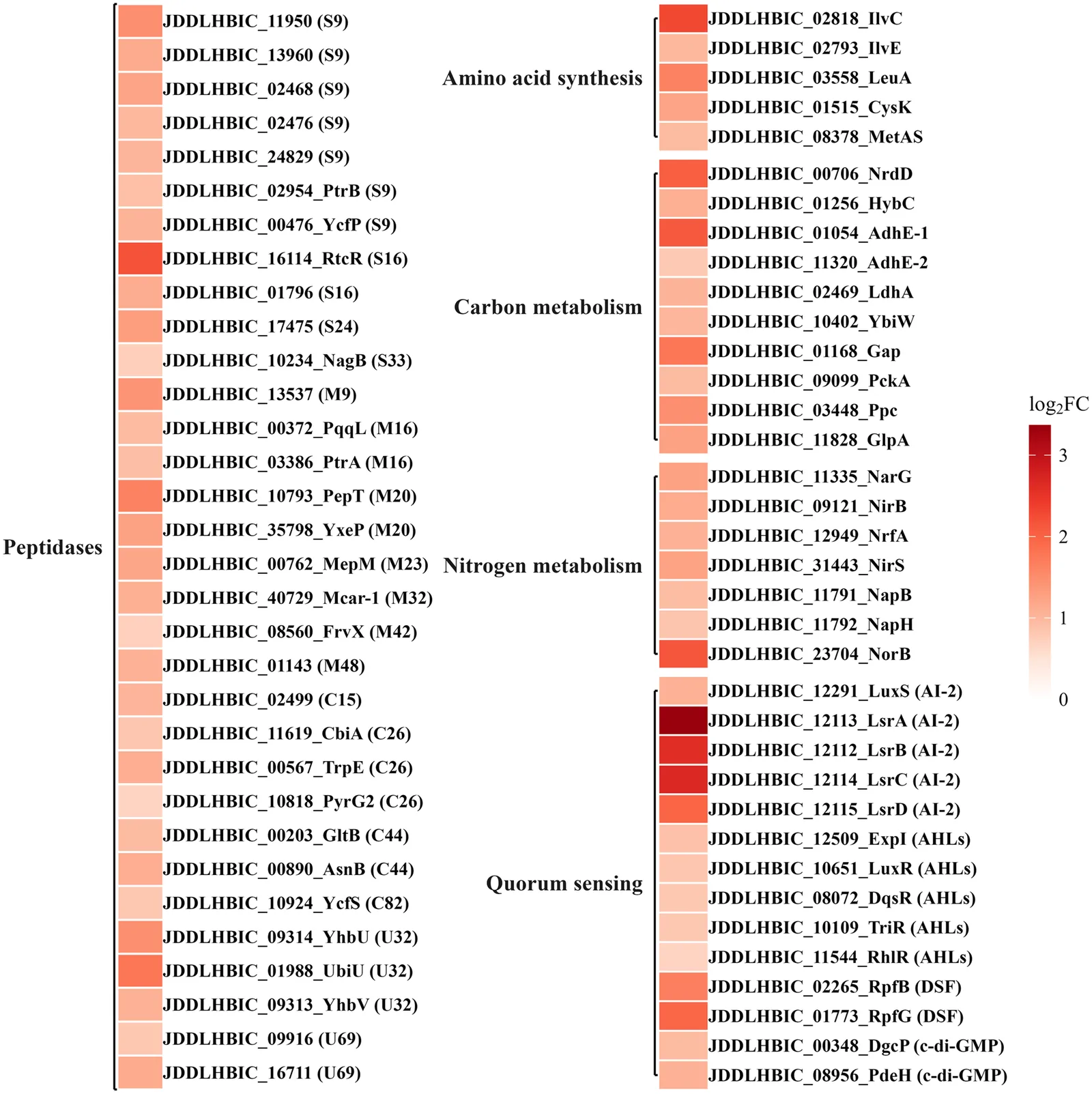
Heatmap of metatranscriptomic differentially expressed genes related to peptidases, carbon metabolism, amino acid biosynthesis, nitrogen metabolism, and quorum sensing.

During MC-RR degradation, 32 peptidase-encoding genes were significantly upregulated, belonging to 17 enzyme families in the MEROPS database and encompassing four major categories: serine peptidases, metallopeptidases, cysteine peptidases, and peptidases of undefined catalytic type (Fig 5). Serine peptidases had the highest number of upregulated genes (families S9, S16, S24, and S33; 4 families,11 genes), with the S9 family contributing 7 upregulated genes including oligopeptidase PtrB and acylaminoacyl-peptidase YcfP. Metallopeptidase families exhibited the greatest diversity (families M20, M23, M9, M16, M32, M42, and M48; 7 families,9 genes), with M23 family metalloendopeptidase MepM, M20 family exopeptidase PepT, and M32 family carboxypeptidase Mcar-1 significantly upregulated. All three U32 family enzymes (YhbU, UbiU, YhbV) were significantly upregulated. Cysteine peptidases involved families C26, C44, C15, and C82, with a total of 7 upregulated genes.

Multiple genes related to nitrogen metabolism, carbon metabolism, and amino acid biosynthesis were significantly upregulated. Among nitrogen metabolism-related genes, nitrate reductase NarG, nitrite reductase NirB, and cytochrome c nitrite reductase NrfA were all significantly upregulated, with NirS, NapB, and NapH also concurrently upregulated; nitric oxide reductase NorB was significantly upregulated. Among energy metabolism-related genes, ribonucleotide reductase NrdD and nickel-iron hydrogenase HybC were significantly upregulated. Among mixed-acid fermentation-related genes, both copies of alcohol dehydrogenase AdhE were upregulated, and lactate dehydrogenase LdhA and pyruvate formate-lyase YbiW were significantly upregulated. Among central carbon metabolism-related genes, glyceraldehyde-3-phosphate dehydrogenase Gap, phosphoenolpyruvate carboxykinase PckA, phosphoenolpyruvate carboxylase Ppc, and glycerol-3-phosphate dehydrogenase GlpA were all significantly upregulated. Among amino acid biosynthesis-related genes, ketol-acid reductoisomerase IlvC of the branched-chain amino acid synthesis pathway showed the highest upregulation, with branched-chain amino acid aminotransferase IlvE and 2-isopropylmalate synthase LeuA concurrently upregulated; in the sulfur-containing amino acid synthesis pathway, cysteine synthase CysK and homoserine O-succinyltransferase MetAS were significantly upregulated. Specific expression changes of these genes are shown in Fig 5.

Multiple quorum sensing (QS) system-related genes were concurrently upregulated (Fig 5). The AI-2 type QS system exhibited the most pronounced transcriptional response, with AI-2 synthase LuxS and its downstream transport system genes LsrA, LsrB, LsrC, and LsrD all substantially upregulated. In the AHLs type QS system, AHLs synthase ExpI and multiple LuxR family transcriptional regulators (LuxR, DqsR, TriR, RhlR) were upregulated. In the DSF type QS system, long-chain fatty acid-CoA ligase RpfB and two-component signal transduction regulator RpfG were significantly upregulated. In the c-di-GMP signaling system, diguanylate cyclase DgcP and phosphodiesterase PdeH were concurrently upregulated.

### Multi-omics-based MC-RR anaerobic degradation pathway and synergistic mechanisms

**Fig 6:**
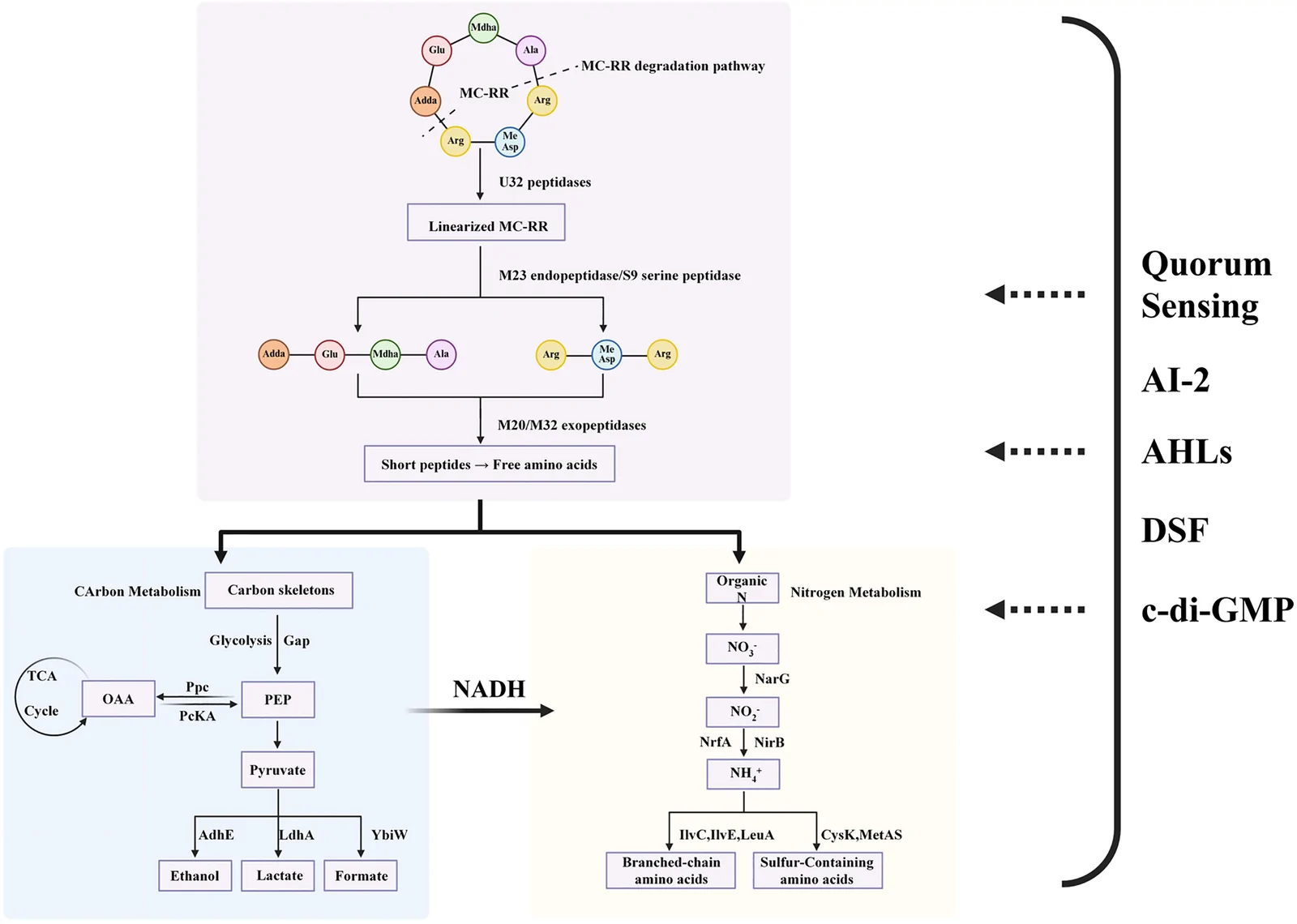
Proposed molecular mechanism of MC-RR by ADMC. This scheme is a conceptual model that integrates LC-MS product identification with metagenomic and metatranscriptomic evidence; transcription-based steps are inferential and require further functional validation.

MAGs functional annotation indicated that *Citrobacter* was enriched in carbohydrate transport and amino acid metabolism-related genes, while *Shewanella* was enriched in energy metabolism and signal transduction-related genes, with emphasis on anaerobic respiration and DNRA-related nitrogen metabolism. *C. amalonaticus* accounted for 76.51% of the community relative abundance and contributed approximately 65% of upregulated DEGs; *S. chilikensis* comprised only 1.36% of the community yet contributed approximately 28% of upregulated genes, revealing a marked asymmetry between relative abundance and transcriptional activity that is consistent with a functional division of labor between the two strains. Based on this functional complementarity, multiple QS systems including AI-2, AHLs, DSF, and c-di-GMP were concurrently upregulated under MC-RR exposure, providing a potential signaling basis for coordinated degradation behavior within the ADMC community. U32 family peptidases were inferred to hydrolyze the Adda-Arg or Ala-Arg bond of MC-RR, achieving ring-opening of the cyclic structure. Subsequently, M23 family metalloendopeptidases, S9 family serine peptidases, and M20/M32 family exopeptidases progressively hydrolyzed the linearized MC-RR into short peptides and free amino acids. The carbon skeleton and organic nitrogen released from enzymatic hydrolysis of MC-RR then entered the central metabolic network, where the community metabolized the carbon skeleton through parallel mixed-acid fermentation and TCA anaplerotic reactions, simultaneously supplying NADH for downstream nitrogen reduction reactions. Upregulation of DNRA-pathway genes suggested that organic nitrogen released from degradation may be reduced to ammonium nitrogen via the NO_3_^−^ → NO_2_^−^ → NH_4_ ^+^ route. The ammonium nitrogen produced by DNRA was further assimilated by the community through branched-chain amino acid synthases and sulfur-containing amino acid synthases, consistent with a potential coupled carbon–nitrogen metabolic linkage (Fig 6). This integrated scheme represents a model proposed from combined metagenomic and metatranscriptomic evidence; the individual enzymatic steps and metabolic couplings remain to be confirmed by direct biochemical, genetic, and biogeochemical measurements.

## Discussion

### Degradation products and candidate peptidases in anaerobic MC-RR transformation

^15^N-labeled LC-MS analysis identified three major ^15^N-containing products in time-series samples: Adda-Glu-Mdha-Ala (m/z 618.3216), its deamination derivative (m/z 600.2965), and Glu-MeAsp-Ala-Arg-CO_2_ (m/z 466.2233). The first two corresponded to an Adda-containing tetrapeptide and its modified product, while the latter corresponded to an Arg-containing tripeptide fragment from the other side of the ring. This product profile suggests that the cyclic structure of MC-RR may have undergone initial cleavage near the Arg-Adda or Ala-Arg bonds, followed by secondary modifications such as deamination. However, several characteristic peaks in the samples remain structurally unassigned; therefore, the degradation pathway is currently better understood as “a multi-step transformation initiated by specific peptide bond cleavage” rather than a fully resolved linear route.

These results are broadly consistent with previous studies on anaerobic MCs degradation. Ding et al. [36] reported through metagenomic analysis that at least three parallel pathways exist for anaerobic MC-LR degradation by enrichment cultures from Lake Taihu; Zhu et al. [22] further identified that MC-LR can undergo ring-opening through hydrolysis of the Ala-Mdha peptide bond. More recently, Yang et al. [37] characterized the anaerobic degradation of MC-LR by *Alcaligenes faecalis* D04 and identified an Adda-Glu-Mdha-Ala-Leu pentapeptide and an Adda-Glu-Mdha tripeptide as major products. Their Adda-Glu-Mdha core is identical to the N-terminal portion of the Adda-Glu-Mdha-Ala tetrapeptide (m/z 618.3216) detected here, suggesting that anaerobic MC degradation across different taxa may converge on a common Adda-proximal peptide intermediate while differing in the exact cleavage position. The putative cleavage sites of MC-RR in the present study (Arg-Adda or Ala-Arg) do not fully coincide with those reported for MC-LR, which may be attributable to differences in amino acid composition at positions 2 and 4—MC-RR contains Arg at both positions, whereas MC-LR contains Leu and Arg, respectively. Such side-chain differences may influence peptidase substrate recognition preferences. This inference, however, requires further support from MC-RR-specific enzymatic data.

At the transcriptomic level, ADMC did not exhibit significant responses of *mlr*-related genes during MC-RR degradation. Instead, 32 peptidase-encoding genes were significantly upregulated, belonging to multiple MEROPS families including U32, M23, S9, M20, and M32. This is consistent with findings from Lake Erie and Lake Taihu sediment samples. Krausfeldt et al. [38] and Mou et al. [39] both reported that MCs degradation in complex natural communities did not always correspond to high expression of the *mlr* gene cluster, and that alternative enzyme systems might be widespread.

Regarding the potential roles of individual peptidase families: all U32 family members were upregulated. This family is known to possess collagenase activity and can recognize proline-rich peptide substrates [40]. Given the rigid cyclic structure and non-proteinogenic amino acid composition of MC-RR, U32 peptidases may be involved in initial substrate recognition or ring-opening, although this inference currently lacks direct enzyme-substrate validation. The S9 family contributed the most upregulated genes (7 genes), including oligopeptidase PtrB and acylaminoacyl-peptidase. This family is known for cleaving peptide bonds at the carboxyl side of proline residues [41], and its upregulation may be related to further cleavage of linearized intermediates. The M23 family, as metalloendopeptidases typically involved in peptidoglycan hydrolysis and specialized peptide substrate cleavage [42], may assist in processing oligopeptide intermediates after ring-opening. The M20 and M32 families are more closely associated with terminal hydrolysis functions and may participate in the final conversion of short peptides to free amino acids. It should also be noted that the functional genes highlighted here (U32, M23, and S9 peptidase families) differ from the hydrolase and degradation genes (e.g., *mblH*, *ridA*, and *paaA*) reported by Yang et al. [37] for anaerobic MC-LR degradation. This divergence likely reflects differences in community composition (a *Citrobacter*–*Shewanella* consortium versus a single *Alcaligenes* isolate) and substrate (MC-RR versus MC-LR), and indicates that anaerobic MC degradation is mediated by taxonomically variable but functionally convergent enzyme systems rather than by a single conserved pathway. Notably, the U32-family member UbiU upregulated in the present study belongs to the same UbiU–UbiV–UbiT system that Yang et al. [37] linked to anaerobic adaptation, offering a shared and testable connection between the two systems.

### Community-level functional differentiation and metabolic complementarity

Metagenomic analysis showed that ADMC was almost entirely composed of Proteobacteria (99.48%) at the phylum level. At the genus level, *Citrobacter* was the dominant taxon (76.51%), followed by *Vibrio* (5.34%), *Enterobacter* (3.47%), and *Shewanella* (1.36%). However, transcriptional contribution analysis revealed a notable asymmetry: although *C. amalonaticus* had the highest abundance and contributed approximately 65% of upregulated differentially expressed genes (DEGs), *S. chilikensis* accounted for only 1.36% of the community yet contributed approximately 28% of upregulated DEGs. Such asymmetry between abundance and transcriptional activity has been reported in other composite degradation systems [30,31], suggesting that low-abundance members may make disproportionate functional contributions at specific metabolic nodes. This study also extends our previous work [33], in which the same ADMC was enriched from shrimp pond sediment and its community succession during MC degradation was described. Whereas that study focused on community enrichment and assembly, the present analysis resolves the degradation products, the non-canonical peptidase repertoire, and the strain-level transcriptional contributions underlying anaerobic MC-RR transformation, thereby adding a mechanistic dimension to those earlier taxonomic observations.

MAG functional annotation further supported the metabolic differences between the two groups. The genome of *C. amalonaticus* was enriched in ABC transporters, PTS systems, glycolysis, and pyruvate metabolism-related genes, consistent with the known characteristics of Enterobacteriaceae in substrate uptake and primary carbon metabolism. The MAGs of the two *Shewanella* strains were more prominent in energy metabolism (oxidative phosphorylation, sulfur metabolism, nitrogen metabolism) and signal transduction (two-component systems). The genus *Shewanella* is well known for its diverse anaerobic respiration capabilities and extracellular electron transfer potential [43], and these genomic features suggest that it may primarily serve functions related to electron acceptor utilization and environmental adaptation in this system.

The significant upregulation of AdhE (alcohol/aldehyde dehydrogenase) and LdhA (lactate dehydrogenase) in the transcriptome indicated that ADMC was in a pronounced mixed-acid fermentation state during MC-RR degradation. This is a typical metabolic mode of Enterobacteriaceae under oxygen-limited conditions, maintaining redox balance by converting organic substrates into small-molecule products such as acetate, lactate, and ethanol [44]. However, fermentative metabolism is often accompanied by product accumulation and thermodynamic constraints [45,46]. In this context, the anaerobic respiration capacity of *Shewanella* may help consume some fermentation products or provide alternative electron acceptor utilization pathways, thereby alleviating product inhibition.

This “fermenter plus respire” combination is not uncommon in anaerobic ecosystems and bears some resemblance to the concept of syntrophy [45,47]. However, it must be noted that the present study only provides associative evidence at the levels of genomic functional annotation and transcriptional response, and has not directly measured metabolite exchange, electron transfer, or material flux between the two groups. Therefore, a more accurate statement would be that *Citrobacter* and *Shewanella* may exhibit a cooperative relationship based on metabolic functional complementarity, but whether this relationship constitutes syntrophy in the strict sense still requires confirmation through co-culture separation experiments, metabolite dynamics monitoring, or isotope flux analysis [45,48].

### Nitrogen metabolism responses and the possibility of carbon-nitrogen coupled utilization

ADMC exhibited pronounced transcriptional responses of nitrogen metabolism-related genes during MC-RR degradation. Nitrate reductase NarG, nitrite reductase NirB, and cytochrome c nitrite reductase NrfA were all significantly upregulated, with NirS, NapB, and NapH also concurrently upregulated. NrfA is a hallmark enzyme of the DNRA (dissimilatory nitrate reduction to ammonium) pathway, and its co-upregulation with NarG and NirB constituted a complete transcriptional response chain of NO_3_^−^ → NO_2_^−^ → NH_4_^+^ at the transcript level.

DNRA is considered an important nitrogen transformation pathway that competes with denitrification in organic carbon-rich, oxygen-limited sediment environments [49,50]. Unlike denitrification, which removes nitrogen from the system as N_2_, DNRA converts nitrate to ammonium, thereby retaining nitrogen within the ecosystem [51]. The transcriptional response of DNRA-related genes in the present study is consistent with this ecological context, suggesting that ADMC may maintain nitrogen retention within the system through DNRA processes while degrading MC-RR.

However, this inference is currently based solely on transcriptomic data. The present study did not simultaneously measure the dynamic changes of nitrate, nitrite, and ammonium concentrations in the culture system, nor did it trace the conversion of organic nitrogen from MC-RR to inorganic nitrogen pools through ^15^N flux analysis. Therefore, at this stage, it can only be stated that ADMC exhibited a transcriptional response pattern consistent with DNRA, rather than confirming that complete DNRA flux conversion has occurred.

The significant upregulation of NorB (nitric oxide reductase) also warrants attention. NorB typically participates in the NO reduction step during denitrification, but in this system, its high expression may be related to a different function: NO is a highly reactive free radical molecule with significant cytotoxicity to bacteria [52]. Organic nitrogen released during MC-RR degradation may generate nitrite or NO intermediates through deamination and oxidation reactions, and the upregulation of NorB may represent a defensive response of the community to nitrogen intermediate-induced stress [53]. However, in the absence of measured NO concentration data, this interpretation remains speculative.

Regarding carbon metabolism, the upregulation of Ppc (phosphoenolpyruvate carboxylase), PckA, and Gap suggested that carbon skeletons released from degradation products may have entered glycolysis and anaplerotic reactions. Concurrently, the significant upregulation of branched-chain amino acid synthesis key enzymes IlvC, IlvE, and LeuA, as well as sulfur-containing amino acid synthesis-related CysK and MetAS, indicated that the community may be actively utilizing nitrogen sources and carbon skeletons released from degradation for biosynthesis [54]. These results collectively suggest that MC-RR removal in this system may not only be a detoxification process but may also be accompanied by reutilization of carbon and nitrogen resources from the toxin molecule. However, it should also be noted that the extent and efficiency of carbon-nitrogen coupled utilization require quantitative data at the metabolic flux level to evaluate.

### Transcriptional responses of quorum sensing-related genes

ADMC also exhibited concurrent upregulation of multiple quorum sensing (QS)-related genes during MC-RR degradation, involving the AI-2, AHLs, DSF, and c-di-GMP signaling systems. Among these, the AI-2 system showed the most prominent response: AI-2 synthase LuxS and its downstream transport system genes LsrA, LsrB, LsrC, and LsrD were all substantially upregulated. In the AHLs system, synthase ExpI and multiple LuxR family transcriptional regulators were also concurrently upregulated. In the DSF system, long-chain fatty acid-CoA ligase RpfB and response regulator RpfG were upregulated. The regulatory role of QS under anaerobic conditions has attracted increasing attention; for example, An et al. [55] showed that manipulating QS through quorum quenching altered enzymatic activities and metabolic pathways in an anaerobic reactor. In the present system, however, the QS-related genes were identified solely from transcriptional co-upregulation, which indicates responsiveness but does not by itself establish a causal regulatory role in MC-RR degradation.

Previous studies provide important references for discussing the role of QS in MCs degradation. Zhou et al. [56] found in an aerobic microbial consortium that the absence of AHL signals reduced MCs degradation efficiency and weakened the expression of related degradation genes, indicating that QS can influence MCs removal at the community level. Zeng et al. [57] further demonstrated in *Novosphingobium* that the LuxR-LuxI type AHL system can promote MCs degradation through transcriptional regulation of the *mlr* gene cluster. These studies were conducted in aerobic or *mlr*-dependent systems, whereas the present study was performed in an anaerobic microbial consortium in which no dominant *mlr* pathway signal was observed. Therefore, whether the upregulation of QS genes in this study directly regulated the MC-RR degradation process cannot be determined at present.

From the perspective of signaling system characteristics, AI-2 is generally considered a cross-species communication signal more closely associated with interspecies coordination [58,59]. The prominent response of the AI-2 system in this study is consistent with the multi-member participation feature of ADMC—in a community composed of members from different genera such as *Citrobacter* and *Shewanella*, the demand for interspecies communication may be higher than in single-strain systems. The upregulation of the Lsr transport system suggests that the community may have enhanced the uptake and processing of extracellular AI-2 signals, which under nutrient-limited or substrate stress conditions may help coordinate the metabolic states of community members [59]. Consistent with this, comparative-genomic work by Chen et al. [60] established that *Shewanella algae*—one of the genomes recovered in this study—harbors the LuxS/AI-2 system together with multiple autoinducer systems, providing a genomic basis for the AI-2 response observed here.

The concurrent upregulation of AHLs and DSF systems suggests that multiple levels of signal regulation may exist within the system. Different types of QS systems may not be functionally independent: AHLs are more involved in intraspecies regulation, while DSF may be related to biofilm formation and metabolic state transitions [61]. The co-response of multiple systems may reflect a comprehensive adaptive strategy of the community when facing complex substrates and anaerobic environments. It should be emphasized that these observations rest on transcript-level co-upregulation; direct evidence, such as QS-signal quantification, exogenous signal supplementation, or QS-inhibition/quorum-quenching assays, would be required to demonstrate that these systems functionally regulate MC-RR degradation. Accordingly, the role of QS in this study is presented as a hypothesis to be tested rather than as an established mechanism.

## Conclusions

This study proposes, based on integrated multi-omics evidence, a candidate anaerobic MC-RR degradation model driven by non-canonical peptidases and DNRA–NorB-associated metabolism in a *Citrobacter*–*Shewanella* consortium. ^15^N labeling confirmed complete MC-RR removal within 3 days, and LC-MS product profiles pointed to initial ring-opening near the Arg-Adda/Ala-Arg bonds. Metagenomics and metatranscriptomics identified U32, M23, and S9 family peptidases as candidate hydrolases, while upregulated *narG*-*nirB*-*nrfA* and *norB* were consistent with DNRA-linked nitrogen turnover and NO detoxification. Multiple QS systems were transcriptionally responsive, and the two dominant taxa exhibited complementary metabolic profiles suggestive of a cooperative “fermentation–respiration” relationship rather than a fully demonstrated syntrophic network. These findings advance understanding of anaerobic MCs attenuation in sediments and support development of in situ bioremediation strategies. Because these conclusions rest largely on metagenomic and metatranscriptomic correlations, targeted biochemical, genetic, and biogeochemical validation will be needed to confirm the proposed enzymatic roles and metabolic couplings.

## Supporting information

S1 Table Semi-preparative HPLC conditions for ^15^N-labeled MC-RR. (XLSX)

S2 Table LC-MS (ESI+) characteristics and time-series peak areas of 20 candidate degradation products from ^15^N MC-RR. (XLSX)

S1 Fig Standard calibration curve for MC-RR. (TIF)

S2 Fig Time-dependent degradation of MC-RR by ADMC. MC-RR concentration was monitored over 72 h with an initial concentration of 1.0 μg/mL. The ADMC-treated group showed progressive degradation of MC-RR over time, whereas the control group without bacterial addition showed no obvious decrease in toxin concentration. Data are presented as mean ± SD from three parallel replicates.
(TIFF)

S3 Fig Functional annotation profiles of high-quality MAG bin5 (*Citrobacter amalonaticus*): (a) COG functional classification, (b) KEGG pathway annotation at level 2, and (c) GO functional annotation at level 2. (TIF)

S4 Fig Functional annotation profiles of high-quality MAG bin12 (*Shewanella chilikensis*): (a) COG functional classification, (b) KEGG pathway annotation at level 2, and (c) GO functional annotation at level 2. (TIF)

S5 Fig Functional annotation profiles of high-quality MAG bin8 (*Shewanella algae*): (a) COG functional classification, (b) KEGG pathway annotation at level 2, and (c) GO functional annotation at level 2. (TIF)
