# Supplementary material for: Putative anaerobic transformation pathway of microcystin-RR inferred from 15N labeling and multi-omics in an enriched shrimp pond sediment microbial community
Source: PLoS One. 2026 Aug 19;21(8):e0355950. doi: 10.1371/journal.pone.0355950 (PMC13489444; doi:10.1371/journal.pone.0355950)
Supplement: Supplementary file 1 [file pone.0355950.s009.docx]

**Response to Reviewers**

**Manuscript ID: PONE-D-26-16301**

**Revised title:** Putative anaerobic transformation pathway of microcystin-RR inferred from ¹⁵N labeling and multi-omics in an enriched shrimp pond sediment microbial community

Dear Editor and Reviewers,

We sincerely thank the Academic Editor and the two reviewers for their careful reading and constructive comments. We have revised the manuscript point by point in response to all comments. In this revision, no new experiments were added; instead, we focused on (i) rigorously moderating claims that exceeded the available evidence, (ii) adding and repositioning key recent literature, (iii) rewriting the Abstract, results synthesis, Discussion, and Conclusions to match the strength of the evidence, and (iv) standardizing figure legends, table headers, molecular-formula formatting, and file presentation. All revised text is highlighted in red in the marked version of the manuscript.

Please note that the “empty sections” and “malformed molecular formulas” noted by the reviewers arose from character/layout dislocation when the submission system converted the source file into the merged PDF. The original .docx file was complete and correctly formatted; we clarify this in the relevant items below and have re-checked the corresponding sections in the revised manuscript.

For clarity, each reviewer comment is quoted below, followed by our response. Newly added references are cited using the numbering of the revised reference list.

**Response to the Academic Editor**

**Editor Comment. Major revision is recommended. Please address all reviewer comments carefully, improve data presentation, and strengthen the discussion.**

**Response:** We have addressed all reviewer comments point by point. Without adding new experiments, we rewrote the Abstract, results synthesis, Discussion, and Conclusions strictly within the boundaries of the available evidence; systematically moderated overstated mechanistic claims; added key recent literature; expanded the methodological rationale; and strengthened the discussion of limitations. We also standardized the figure legends, table headers, molecular-formula formatting, and the description of Fig 6 to improve the readability and internal consistency of the manuscript.

**Response to Journal Requirements**

**1. Please ensure that your manuscript meets PLOS ONE style requirements, including those for file naming.**

**Response:** We have checked and adjusted the manuscript against the PLOS ONE main-body and title/authors/affiliations templates. Figures, tables, and supporting information are numbered and named according to PLOS ONE conventions (Fig 1–6, Table 1, S1–S2 Table, S1–S5 Fig), and the submission files are named as required.

**2. We note that the grant information provided in the Funding Information and Financial Disclosure sections do not match. Please provide the correct grant numbers in the Funding Information section.**

**Response:** We have re-checked the funding information. The awards and grant numbers for this study are as follows, and we will update the online Funding Information section accordingly so that it fully matches the Financial Disclosure: National Natural Science Foundation of China (No. 32172978); Natural Science Foundation of Tianjin (25JCYBJC00800); Tianjin Science and Technology Planning Project (24ZYCGSN00140); Gansu Science and Technology Project (No. 24CXNP011 and 24CXNP005); and the Innovation Training Program Project for College Students of Tianjin Agricultural University (No. 202410061160).

**3. Financial disclosure – funder role and author affiliation with the funder.**

**Response:** We confirm that the funders provided financial support only and had no role in study design, data collection and analysis, decision to publish, or preparation of the manuscript. We will confirm the corresponding statement in the cover letter and will accurately complete the author-contributions information in the online form. The suggested financial-disclosure statement is: “This work was supported by the National Natural Science Foundation of China (No. 32172978), the Natural Science Foundation of Tianjin (25JCYBJC00800), the Tianjin Science and Technology Planning Project (24ZYCGSN00140), the Gansu Science and Technology Project (No. 24CXNP011 and 24CXNP005), and the Innovation Training Program Project for College Students of Tianjin Agricultural University (No. 202410061160). The funders had no role in study design, data collection and analysis, decision to publish, or preparation of the manuscript.”

**4. Please amend the manuscript submission data to include authors Jingjing Zhang, Rui Chen, Wenjie Zhao, Dajuan Zhang, Qian Wang, and Xinyu Wang.**

**Response:** The complete author list has been confirmed in the manuscript. We will update the author information in the online submission system via Edit Submission to include Jingjing Zhang, Rui Chen, Wenjie Zhao, Dajuan Zhang, Qian Wang, and Xinyu Wang, so that the system record matches the manuscript.

**5. If the reviewer comments include a recommendation to cite specific previously published works, please review and evaluate these publications for relevance.**

**Response:** We have evaluated the works suggested by the reviewers and consider them directly relevant; they have been cited accordingly: Yang et al. (2025) [37], An et al. (2026) [55], and Chen et al. (2020) [60]. The reference list has been renumbered in order of first appearance in the text, and all in-text citations correspond consistently to the final list.

**Response to Reviewer #1**

We thank the reviewer for recognizing the value of integrating isotope tracing with multi-omics and for the constructive concerns raised. We respond to each point below.

**Comment 1. The manuscript heavily relies on correlations between metatranscriptomic upregulation and inferred enzymatic functions to propose a novel anaerobic degradation pathway. No direct biochemical or genetic validation is provided to confirm the roles of U32, M23, or S9 peptidases. The conclusions should be supported with functional evidence.**

**Response:** We fully agree. As functional validation experiments (enzyme assays, gene knockouts, or heterologous expression) are beyond the scope of the present study, we have moderated the corresponding claims to match the available evidence and now explicitly frame them as candidate hypotheses derived from multi-omics associations rather than established mechanisms. Specifically: (i) in the Abstract, “constituting an independent anaerobic degradation pathway” was changed to “suggesting the involvement of a non-canonical, mlr-independent peptidase system in MC-RR transformation”; (ii) in the Results and Conclusions, the peptidases are consistently described as “candidate hydrolases,” with an added statement that the inferred enzymatic roles require targeted biochemical, genetic, and biogeochemical validation; and (iii) the qualifying statement that the inference lacks direct enzyme–substrate validation has been retained and emphasized in the Discussion. Heterologous expression and in vitro enzyme assays have been identified as clear next steps.

**Comment 2. Only three products are tentatively assigned and many peaks remain unresolved. The proposed pathway is incomplete and lacks structural confirmation. Inferring specific cleavage sites (Arg–Adda or Ala–Arg) is not sufficiently convincing.**

**Response:** We agree. We now clarify that product assignments are based on accurate-mass (MS1) matching within ±5 ppm and are therefore tentative, because the MS/MS fragmentation and NMR data required for unambiguous confirmation of the cleavage sites were not obtained in this study. The cleavage sites are now described as “tentatively inferred,” and the process is presented as “a multi-step transformation initiated by specific peptide-bond cleavage” rather than a fully resolved linear pathway. The three key products are labeled “tentative” in the Abstract, and the full set of 20 candidate peaks (mass, retention time, and time-series peak areas) is provided in S2 Table.

**Comment 3. The claim of DNRA activation and carbon–nitrogen coupling is based solely on gene expression data, without measurement of nitrogen species or isotope tracing of nitrogen flux.**

**Response:** We accept this criticism and have systematically moderated the relevant statements. (i) In the Abstract, the definitive statements on DNRA activation, conversion of organic nitrogen into recoverable ammonium, and efficient carbon–nitrogen coupling were replaced with “narG–nirB–nrfA and norB were concurrently upregulated, a transcriptional pattern consistent with DNRA-linked nitrogen turnover and NO detoxification during degradation, although the corresponding nitrogen fluxes were not directly measured.” (ii) In the Results, “the DNRA pathway reduced organic nitrogen to ammonium” was changed to “upregulation of DNRA-pathway genes suggested that organic nitrogen may be reduced to ammonium,” with a statement that the integrated scheme is a model requiring biogeochemical confirmation. (iii) The Discussion explicitly states that nitrate, nitrite, and ammonium dynamics were not measured and that no ¹⁵N nitrogen-flux tracing was performed; therefore, only a transcriptional response consistent with DNRA can be claimed, not confirmed DNRA flux.

**Comment 4. The initial MC-RR concentration (1 microgram/mL) is not representative of most natural environments, raising concerns about ecological relevance and scalability.**

**Response:** We have added the rationale for this concentration in the Materials and methods. Although higher than levels typically encountered in ambient surface waters, 1 µg/mL was deliberately selected to ensure sufficient accumulation of transient degradation intermediates for reliable LC-MS detection and pathway reconstruction, consistent with previous mechanistic studies of MC biodegradation [19,22]. We also note that intracellular MC released during bloom collapse can substantially elevate local concentrations at the sediment–water interface. As the present study focuses on mechanistic resolution rather than quantitative degradation kinetics, degradation efficiency at environmentally realistic concentrations will be examined in future work, and we have moderated statements regarding direct application value accordingly.

**Comment 5. The role of QS under anaerobic conditions (Bioresour. Technol., 2026, 442, 133668) should be covered. The manuscript attributes a central regulatory role to multiple QS systems without experimental validation; this section is largely speculative and should be reduced or supported.**

**Response:** We thank the reviewer for this key reference, which we have cited (An et al., Bioresour. Technol. 2026, 442, 133668 [55]). We note that the regulatory role of QS under anaerobic conditions has attracted increasing attention; for example, An et al. showed that manipulating QS through quorum quenching altered enzymatic activities and metabolic pathways in an anaerobic reactor. We have also substantially reduced the speculative framing of this section: we now state that the QS-related genes were identified solely from transcriptional co-upregulation, which indicates responsiveness but does not establish a causal regulatory role, and that QS-signal quantification, exogenous signal supplementation, or QS-inhibition/quorum-quenching assays would be required to demonstrate functional regulation. The role of QS is therefore presented as a hypothesis to be tested. The phrase “multi-layered regulatory network ensuring precise division of labor” has been removed from the Abstract.

**Comment 6. The proposed syntrophic fermentation–respiration network between Citrobacter and Shewanella is inferred solely from annotation and transcriptional contribution, without co-culture, metabolite-exchange, or electron-transfer analyses.**

**Response:** We agree and have removed the strict syntrophy designation. In the Conclusions, “establishing a syntrophic fermentation–respiration network” was changed to “the two dominant taxa exhibited complementary metabolic profiles suggestive of a cooperative fermentation–respiration relationship rather than a fully demonstrated syntrophic network.” The Discussion retains the explicit statement that only genome-annotation and transcriptional-response evidence is available and that metabolite exchange, electron transfer, and material flux were not measured; therefore, whether the relationship constitutes syntrophy in the strict sense requires confirmation by co-culture, metabolite monitoring, or isotope-flux analysis. “Precise division of labor” has been replaced by a neutral description of the low-abundance but transcriptionally active taxon.

**Comment 7. Similar observations (mlr-independent degradation, diverse peptidases, community-level degradation) have been reported previously. The manuscript does not clearly delineate how its findings advance beyond prior studies.**

**Response:** We have moderated the novelty claims (removing “novel mechanism” from the Abstract and Conclusions and replacing it with “a candidate model”) and now explicitly define the incremental contribution of this study rather than claiming a new pathway. mlr-independent and community-level degradation have indeed been reported; our incremental contribution is: (i) using ¹⁵N labeling combined with time-series LC-MS to provide product-level evidence for anaerobic MC-RR (rather than the more commonly studied MC-LR) degradation; (ii) integrating metagenomic binning with metatranscriptomics to match degradation-related candidate peptidases (U32/M23/S9 and other MEROPS families) with specific taxa and to resolve strain-level transcriptional contributions (S. chilikensis, at only 1.36% abundance, contributed approximately 28% of upregulated genes); and (iii) positioning these results against the most recent work, including Yang et al. (2025) [37]. We now state that different taxa use different functional genes but converge functionally, and we do not claim a single universal conserved pathway.

**Comment 8. The manuscript frequently uses strong claims (novel mechanism, precise division of labor, efficient carbon–nitrogen coupling) that are not fully supported.**

**Response:** We fully agree and have revised these expressions throughout: (i) “novel mechanism” to “a candidate model”; (ii) “precise division of labor” to “functional division of labor / complementary metabolic profiles,” with the phrase “ensuring precise division of labor” deleted; (iii) “efficient carbon–nitrogen metabolic coupling” to “a transcriptional pattern consistent with … / a potential coupled carbon–nitrogen linkage”; and (iv) “activated” to “concurrently upregulated.” These changes are concentrated in the Abstract, Results, and Conclusions.

**Comment 9. The abstract is overly dense and contains excessive mechanistic claims not fully supported in the main text.**

**Response:** The Abstract has been rewritten. We removed unsupported mechanistic assertions (“constituting an independent pathway,” “the DNRA pathway was activated … achieving efficient carbon–nitrogen coupling,” and “a multi-layered regulatory network ensuring precise division of labor”), added “tentative” to the product assignments, and revised the descriptions of the peptidases, DNRA, and QS to evidence-matched wording (“suggesting/consistent with/concurrently upregulated”), while adding the key qualifier that nitrogen fluxes were not directly measured. The revised Abstract retains the core findings while markedly reducing the density and strength of the claims.

**Comment 10. Figures (especially pathway diagrams) are conceptual and lack quantitative support.**

**Response:** We agree that Fig 6 is essentially an integrative conceptual model. To avoid misinterpretation, we added a clarifying statement to the Fig 6 legend: the scheme is a conceptual model integrating LC-MS product identification with metagenomic and metatranscriptomic evidence, and the transcription-based steps are inferential and require further functional validation. We note that the quantitative differential-expression information underlying the model is provided by Fig 5 (heatmap of DEGs for peptidases, carbon/nitrogen metabolism, amino-acid biosynthesis, and QS) and Fig 4 (volcano plot); Fig 6 is used to present the metabolic logic suggested by these quantitative results and is referred to as a proposed/conceptual model in the text.

**Response to Reviewer #2**

We thank the reviewer for the positive assessment and for the valuable suggestions on contextualizing our findings against recent 2024/2025 studies. We respond to each point below.

**Comment 1. The manuscript fails to engage with critical contemporary literature such as Yang et al. (2025), which identifies a different set of functional genes and pathways for anaerobic MC degradation.**

**Response:** We have cited Yang et al. (2025, Ecotoxicol. Environ. Saf. 290:117721 [37]) and added a comparison in the Discussion. Yang et al. reported anaerobic MC-LR degradation by Alcaligenes faecalis D04, identifying an Adda-Glu-Mdha-Ala-Leu pentapeptide and an Adda-Glu-Mdha tripeptide and linking mblH, ridA, and paaA to the degradation function; their functional gene set differs from the U32/M23/S9 peptidase system reported here. Accordingly, we have moderated claims of universality/novelty and now state that anaerobic MC degradation is mediated by taxonomically variable but functionally convergent enzyme systems rather than a single conserved pathway. We also note a testable connection: the Adda-Glu-Mdha core of the products reported by Yang et al. is identical to the N-terminal portion of our Adda-Glu-Mdha-Ala tetrapeptide (m/z 618.3216), suggesting convergence on a common Adda-proximal intermediate, and the U32-family member UbiU upregulated here belongs to the same UbiU–UbiV–UbiT system that Yang et al. linked to anaerobic adaptation.

**Comment 2. The novelty of identifying Shewanella as a key anaerobic MC degrader in shrimp pond sediments is diminished by a failure to reference Zhao et al. (2025), which reports nearly identical findings in the same ecological niche.**

**Response:** We clarify that Zhao et al. (2025) is prior work from our own group and was already cited in the original manuscript as reference [33] (Zhao W, Bi X, Wang X, Liu C, Zhang D, Dai W. Water. 2025;17(6):774). To make the relationship explicit, we added a distinction in the Discussion: the earlier work [33] focused on anaerobic enrichment and community succession of the ADMC, whereas the present study further resolves the degradation products, the non-canonical peptidase repertoire, and strain-level transcriptional contributions, thereby adding a mechanistic dimension to the earlier taxonomic observations. This study therefore advances the same system from community composition to functional mechanism rather than repeating the identification of Shewanella.

**Comment 3. Major portions of the manuscript, including the Introduction and Materials and methods, are currently empty or contain only submission-template instructions.**

**Response:** We apologize for the inconvenience. On inspection, the Introduction and Materials and methods in the original .docx file were complete, covering the background on eutrophication and MC, the status of anaerobic degradation research, the multi-omics and isotope-labeling strategy, and full methodological descriptions (ADMC source, ¹⁵N-MC-RR preparation, degradation-product analysis, nucleic-acid extraction and sequencing, metagenomic/metatranscriptomic analyses, and multi-omics integration). The empty/template-only appearance resulted from the submission system failing to embed these sections correctly when generating the merged PDF, not from any omission in the manuscript. We have re-verified that these sections are complete and will submit properly formatted files to avoid such conversion issues.

**Comment 4. The Results section contains malformed molecular formulas (e.g., separating element counts from symbols) and tables missing clear column headers.**

**Response:** On inspection, the molecular formulas in the original .docx used correct superscript/subscript formatting (e.g., C₃₂H₄₆¹⁵N₄O₈); the dislocations seen by the reviewer (e.g., C H15N2O7 17 34) were likewise caused by superscript/subscript characters being rearranged during PDF merging, not by errors in the source text. We have re-checked all superscript/subscript formatting in the Results and provide a corrected, standard list of ¹⁵N intermediates below (Reviewer #2, Question 3). The column headers of S2 Table (Mass, RT, peak area, etc.) were complete in the original file, and we have confirmed that they are clear.

**Comment 5. The theoretical framing of the QS and DNRA results lacks grounding in established literature, e.g., failing to cite prior work on AI-2 regulatory patterns in the Shewanella genus.**

**Response:** We have added the relevant literature and strengthened the theoretical framing: (i) for AI-2 regulation in the genus Shewanella, we now cite Chen et al. (2020, Comp. Immunol. Microbiol. Infect. Dis. 73:101545 [60]), a comparative-genomics study establishing that Shewanella algae — one of the genomes recovered in this study — harbors the LuxS/AI-2 system and multiple autoinducer systems, providing a genomic basis for the observed AI-2 response; (ii) for QS regulation under anaerobic conditions, we now cite An et al. (2026) [55]; and (iii) the DNRA discussion continues to rest on the narG–nirB–nrfA transcriptional-response chain, retaining prior literature on the competition between DNRA and denitrification in carbon-rich, oxygen-limited sediments [49–51]. The evidential boundaries of these interpretations have been clarified as noted for Reviewer #1.

**Question 1. How do the non-canonical peptidases (U32, M23, S9 families) reconcile with the alternative anaerobic degradation genes (mblH, ridA, paaA) and intermediates identified by Yang et al. (2025)?**

**Response:** Our view is functional convergence with variable genes, now addressed explicitly in the Discussion. (i) At the product level, the pentapeptide/tripeptide products of Yang et al. share the Adda-Glu-Mdha core, identical to the N-terminus of our Adda-Glu-Mdha-Ala tetrapeptide (m/z 618.3216), suggesting that different anaerobic systems may converge on a common Adda-proximal peptide intermediate while differing in the exact cleavage position (Arg-Adda/Ala-Arg here versus the corresponding MC-LR sites in Yang et al.). (ii) At the gene level, the difference between the two systems (U32/M23/S9 versus mblH/ridA/paaA) likely reflects differences in community composition (a Citrobacter–Shewanella consortium versus a single Alcaligenes isolate) and substrate (MC-RR versus MC-LR), indicating that anaerobic MC degradation does not rely on a single conserved pathway. (iii) A specific testable link exists: the U32-family member UbiU upregulated here belongs to the anaerobic-adaptation-related UbiU–UbiV–UbiT system described by Yang et al., providing an entry point for future cross-system comparison.

**Question 2. What specific novel mechanistic insights does this study provide regarding the Citrobacter–Shewanella synergy that differentiate it from Zhao et al. (2025)?**

**Response:** As noted above, Zhao et al. (2025) [33] is prior work from our group focused on anaerobic enrichment and community succession of the ADMC (i.e., which taxa are present and how they succeed). The mechanistic increment of the present study is: (i) providing product-level evidence and candidate cleavage sites for anaerobic MC-RR degradation through ¹⁵N labeling plus time-series LC-MS; (ii) matching candidate peptidases (U32/M23/S9, etc.) to degradation products through binning plus metatranscriptomics, and quantifying strain-level transcriptional contributions — revealing a marked asymmetry between abundance and transcriptional activity (S. chilikensis, 1.36% abundance, approximately 28% of upregulated genes) that points to functional roles of low-abundance members at specific metabolic nodes; and (iii) characterizing the metabolic-complementarity-based cooperative relationship between Citrobacter (substrate uptake and primary carbon metabolism) and Shewanella (anaerobic respiration/electron-acceptor utilization and signal transduction). This study therefore advances the system from community composition to functional mechanism.

**Question 3. Can the authors clarify the molecular-formula formatting issues (e.g., C H15N2O7 17 34) and provide a corrected, standard list of identified 15N intermediates?**

**Response:** Yes. As noted, the dislocation was caused by PDF conversion; the source .docx uses correct superscript/subscript formatting. Below is the corrected, standard list of the nine candidate ¹⁵N-containing intermediates (subscripts denote atom counts; ¹⁵N denotes ¹⁵N-labeled nitrogen):

m/z 618.3216 → C₃₂H₄₆¹⁵N₄O₈	(Product 1: Adda-Glu-Mdha-Ala)

m/z 600.2965 → C₃₂H₄₃¹⁵N₃O₈	(Product 2: deamination product)

m/z 466.2233 → C₁₈H₃₃¹⁵N₇O₇	(Product 3: Glu-MeAsp-Ala-Arg-CO₂)

m/z 855.6404 → C₄₅H₈₅¹⁵N₁₀O₅

m/z 380.2312 → C₁₇H₃₄¹⁵N₂O₇

m/z 279.1833 → C₁₂H₂₆¹⁵N₃O₄

m/z 352.1166 → C₇H₂₀¹⁵N₈O₈

m/z 603.3989 → C₂₇H₅₈¹⁵N₃O₁₁

m/z 506.3468 → C₂₅H₄₉¹⁵N₃O₇

The remaining 11 characteristic peaks yielded no matching molecular formula within the specified elemental range. The complete set of all 20 candidate peaks (mass, retention time, and time-series peak areas) is listed in S2 Table.

**Question 4. Could the authors provide a version of the manuscript that populates the currently empty Introduction and Materials and Methods sections and removes the editorial placeholders and instructional text?**

**Response:** Yes. As noted, these sections were complete in the original manuscript and contained no placeholder text; the issue arose during PDF merging. We submit properly formatted, complete files (a marked version and a clean copy) in which the Introduction and Materials and Methods are fully presented and free of any template/placeholder text.

We thank the Editor and the two reviewers again for their valuable comments. We believe that these revisions have substantially improved the rigor of the manuscript and the clarity of its evidential boundaries. We look forward to your further feedback.

Sincerely,

Xiangdong Bi and Wei Dai, on behalf of all authors
