# Supplementary material for: Putative anaerobic transformation pathway of microcystin-RR inferred from 15N labeling and multi-omics in an enriched shrimp pond sediment microbial community
Source: PLoS One. 2026 Aug 19;21(8):e0355950. doi: 10.1371/journal.pone.0355950 (PMC13489444; doi:10.1371/journal.pone.0355950)
Supplement: Peer review history

# Peer review: Decision Letter 0

**DOI:** 10.1371/journal.pone.0355950.r001

**Authors:** Bijay Kumar Behera

24 May 2026

Dear Dr. liu,

Thank you for submitting your manuscript to PLOS ONE. After careful consideration, we feel that it has merit but does not fully meet PLOS ONE’s publication criteria as it currently stands. Therefore, we invite you to submit a revised version of the manuscript that addresses the points raised during the review process.

Please submit your revised manuscript by Jul 08 2026 11:59PM. If you will need more time than this to complete your revisions, please reply to this message or contact the journal office at plosone@plos.org . When you're ready to submit your revision, log on to https://www.editorialmanager.com/pone/ and select the 'Submissions Needing Revision' folder to locate your manuscript file.

- A letter that responds to each point raised by the academic editor and reviewer(s). You should upload this letter as a separate file labeled 'Response to Reviewers'.
- A marked-up copy of your manuscript that highlights changes made to the original version. You should upload this as a separate file labeled 'Revised Manuscript with Track Changes'.
- An unmarked version of your revised paper without tracked changes. You should upload this as a separate file labeled 'Manuscript'.

If you would like to make changes to your financial disclosure, please include your updated statement in your cover letter. Guidelines for resubmitting your figure files are available below the reviewer comments at the end of this letter.

If applicable, we recommend that you deposit your laboratory protocols in protocols.io to enhance the reproducibility of your results. Protocols.io assigns your protocol its own identifier (DOI) so that it can be cited independently in the future. For instructions see: https://journals.plos.org/plosone/s/submission-guidelines#loc-laboratory-protocols . Additionally, PLOS ONE offers an option for publishing peer-reviewed Lab Protocol articles, which describe protocols hosted on protocols.io. Read more information on sharing protocols at https://plos.org/protocols?utm_medium=editorial-email&utm_source=authorletters&utm_campaign=protocols .

As the corresponding author, your ORCID iD is verified in the submission system and will appear in the published article. PLOS supports the use of ORCID, and we encourage all coauthors to register for an ORCID iD and use it as well. Please encourage your coauthors to verify their ORCID iD within the submission system before final acceptance, as unverified ORCID iDs will not appear in the published article. *Only*   the individual author can complete the verification step; PLOS staff *cannot*   verify ORCID iDs on behalf of authors.

We look forward to receiving your revised manuscript.

Kind regards,

Bijay Kumar Behera, Ph.D.

Academic Editor

PLOS One

Journal Requirements:

When submitting your revision, we need you to address these additional requirements.

1. Please ensure that your manuscript meets PLOS ONE's style requirements, including those for file naming. The PLOS ONE style templates can be found at

https://journals.plos.org/plosone/s/file?id=wjVg/PLOSOne_formatting_sample_main_body.pdf and

https://journals.plos.org/plosone/s/file?id=ba62/PLOSOne_formatting_sample_title_authors_affiliations.pdf

2. We note that the grant information you provided in the ‘Funding Information’ and ‘Financial Disclosure’ sections do not match.

When you resubmit, please ensure that you provide the correct grant numbers for the awards you received for your study in the ‘Funding Information’ section.

3. Thank you for stating the following financial disclosure:

“This work was supported by the National Natural Science Foundation of China (grant No. 32172978 to XB; https://www.nsfc.gov.cn/english/site_1/index.html), the Natural Science Foundation of Tianjin (grant No. 25JCYBJC00800 to XB; https://kjgh.tj.gov.cn/), the Tianjin Science and Technology Planning Project (grant No. 24ZYCGSN00140 to XB; https://kjgh.tj.gov.cn/), the Gansu Science and Technology Project (grant Nos. 24CXNP011 and 24CXNP005 to XB; https://kjt.gansu.gov.cn/), and the Innovation Training Program Project for College Students of Tianjin Agricultural University (grant No. 202410061160 to XB; https://www.tjau.edu.cn/). The funders had no role in study design, data collection and analysis, decision to publish, or preparation of the manuscript.”

We note that one or more of the authors is affiliated with the funding organization, indicating the funder may have had some role in the design, data collection, analysis or preparation of your manuscript for publication; in other words, the funder played an indirect role through the participation of the co-authors. If the funding organization did not play a role in the study design, data collection and analysis, decision to publish, or preparation of the manuscript and only provided financial support in the form of authors' salaries and/or research materials, please do the following:

1. Review your statements relating to the author contributions, and ensure you have specifically and accurately indicated the role(s) that these authors had in your study. These amendments should be made in the online form.

2. Confirm in your cover letter that you agree with the following statement, and we will change the online submission form on your behalf:

“The funder provided support in the form of salaries for authors [insert relevant initials], but did not have any additional role in the study design, data collection and analysis, decision to publish, or preparation of the manuscript. The specific roles of these authors are articulated in the ‘author contributions’ section.

4. Please amend the manuscript submission data (via Edit Submission) to include author Jingjing Zhang, Rui Chen, Wenjie Zhao, Dajuan Zhang, Qian Wang and Xinyu Wang.

5. If the reviewer comments include a recommendation to cite specific previously published works, please review and evaluate these publications to determine whether they are relevant and should be cited. There is no requirement to cite these works unless the editor has indicated otherwise.

Additional Editor Comments:

After careful evaluation of the manuscript entitled “Mechanism of anaerobic microcystin (MC) degradation by microbial community enriched in shrimp pond sediment”, and considering the comments provided by the reviewers, I recommend a Major Revision before the manuscript can be considered further for publication.

The study addresses an important topic related to anaerobic degradation of microcystin by sediment-associated microbial communities and has potential scientific significance in the field of environmental microbiology and bioremediation. The experimental approach and overall findings are promising. However, several important concerns regarding methodological clarity, data interpretation, presentation of results, and discussion need to be adequately addressed.

Reviewer 1 has raised substantial issues that require detailed revision and clarification, particularly regarding experimental design, mechanistic interpretation, microbial community analysis, and supporting evidence for the proposed degradation pathways. Reviewer 2 has also provided valuable suggestions for improving the manuscript presentation and scientific clarity.

The authors are therefore requested to carefully address all reviewer comments point-by-point and revise the manuscript accordingly. Additional clarification, improved data presentation, and strengthening of the discussion section will significantly enhance the overall quality and impact of the manuscript.

[Note: HTML markup is below. Please do not edit.]

Reviewers' comments:

Reviewer's Responses to Questions

**Comments to the Author**

1. Is the manuscript technically sound, and do the data support the conclusions?

Reviewer #1: Yes

Reviewer #2: Yes

**********

2. Has the statistical analysis been performed appropriately and rigorously?

Reviewer #1: Yes

Reviewer #2: Yes

**********

3. Have the authors made all data underlying the findings in their manuscript fully available?

The PLOS Data policy

Reviewer #1: Yes

Reviewer #2: Yes

**********

4. Is the manuscript presented in an intelligible fashion and written in standard English?

Reviewer #1: Yes

Reviewer #2: Yes

**********

Reviewer #1: This manuscript investigates the anaerobic degradation mechanism of microcystin-RR (MC-RR) by a microbial community enriched from shrimp pond sediments, integrating LC–MS/MS with metagenomic and metatranscriptomic analyses. The integration of isotope tracing with multi-omics provides a potentially valuable framework for understanding anaerobic toxin attenuation in sediment environments, and the findings may have practical implications for developing in situ bioremediation strategies targeting cyanotoxins. However, I have major concerns that should be addressed before this paper can be considered for publication.

1. The manuscript heavily relies on correlations between metatranscriptomic upregulation and inferred enzymatic functions to propose a “novel anaerobic degradation pathway.” However, no direct biochemical or genetic validation (e.g., enzyme assays, gene knockouts, or heterologous expression) is provided to confirm the roles of U32, M23, or S9 peptidases in MC-RR degradation. The conclusions should be supported with functional evidence.

2. Although LC-MS identifies multiple candidate products, only three are tentatively assigned, and many peaks remain unresolved. The proposed degradation pathway is therefore incomplete and lacks structural confirmation (e.g., MS/MS fragmentation patterns, NMR validation). Inferring specific cleavage sites (Arg–Adda or Ala–Arg) based on limited evidence is not sufficiently convincing and may be misleading.

3. The authors claim activation of the DNRA pathway and carbon–nitrogen coupling; however, this conclusion is based solely on gene expression data. No measurements of nitrogen species (NO₃⁻, NO₂⁻, NH₄⁺) or isotope tracing of nitrogen flux are provided. Without quantitative biogeochemical data, the assertion of DNRA-driven nitrogen recycling is speculative and overextended.

4. the initial MC-RR concentration (1 μg/mL) is not representative of most natural environments. This raises concerns about the ecological relevance and scalability of the findings. The absence of comparison with environmental concentrations undermines the claimed application value.

5. The role of QS in the anaerobic conditions Bioresour. Technol., 2026, 442, 133668 should be covered. The manuscript attributes a central regulatory role to multiple QS systems (AI-2, AHLs, DSF, c-di-GMP), yet provides no experimental validation (e.g., QS inhibition, signal quantification, or gene knockout). The observed transcriptional upregulation does not demonstrate functional involvement in MC-RR degradation. This section is largely speculative and should be significantly reduced or experimentally supported.

6. The authors propose a “syntrophic fermentation–respiration network” between Citrobacter and Shewanella, but this is inferred solely from genomic annotation and transcriptional contribution. No co-culture experiments, metabolite exchange measurements, or electron transfer analyses are conducted. The claim of syntrophy is therefore not justified and reflects conceptual overreach.

7. Although the study emphasizes a “novel anaerobic pathway,” similar observations, such as mlr-independent degradation, involvement of diverse peptidases, and community-level degradation, have been reported previously. The manuscript does not clearly delineate how its findings fundamentally advance beyond prior studies, particularly in terms of mechanistic resolution.

8. The manuscript frequently uses strong claims such as “novel mechanism,” “precise division of labor,” and “efficient carbon–nitrogen coupling,” which are not fully supported by the data. This rhetorical inflation detracts from scientific credibility and should be revised to reflect actual evidence.

9. The abstract is overly dense and contains excessive mechanistic claims not fully supported in the main text.

10. Figures (especially pathway diagrams) are conceptual and lack quantitative support.

Reviewer #2: This manuscript investigates the anaerobic degradation of Microcystin-RR in shrimp pond sediments using a combination of stable isotope labeling and multi-omics to propose a synergistic metabolic pathway between Citrobacter and Shewanella. While the technical approach is sophisticated and the findings regarding non-canonical enzyme systems are intriguing, the manuscript required revisions before publication and contextualize the findings against several very recent 2024/2025 studies that propose alternative anaerobic pathways and taxonomic roles in the same ecological niche.

− The manuscript fails to engage with critical contemporary literature, such as Yang et al. (2025), which identifies a different set of functional genes and pathways for anaerobic MC degradation, potentially overstating the novelty or universality of the proposed peptidase system.

− The novelty of identifying Shewanella as a key anaerobic microcystin degrader in shrimp pond sediments is diminished by a failure to reference Zhao et al. (2025), which reports nearly identical findings in the same ecological niche.

− Major portions of the manuscript, including the Introduction and Materials and methods, are currently empty or contain only submission template instructions rather than scientific content.

− The Results section contains malformed molecular formulas (e.g., separating element counts from symbols) and tables missing clear column headers, which obscures the presentation of the core chemical data.

− The theoretical framing of the quorum sensing and DNRA results lacks grounding in established literature, such as failing to cite prior work establishing the AI-2 regulatory patterns in the Shewanella genus.

− How do the identified non-canonical peptidases (U32, M23, S9 families) reconcile with the alternative anaerobic degradation genes (mblH, ridA, paaA) and intermediates identified by Yang et al. (2025)?

− What specific novel mechanistic insights does this study provide regarding the Citrobacter–Shewanella synergy that differentiate it from the recently published work by Zhao et al. (2025), which also identified Shewanella as a key anaerobic MC-degrader in shrimp pond sediments?

− Can the authors clarify the molecular formula formatting issues in the Results section (e.g., C H15N2O7 17 34) and provide a corrected, standard list of identified 15N intermediates?

− Could the authors provide a version of the manuscript that populates the currently empty Introduction and Materials and Methods sections and removes the editorial placeholders and instructional text?

**********

what does this mean? ). If published, this will include your full peer review and any attached files.

If you choose “no”, your identity will remain anonymous but your review may still be made public.

**Do you want your identity to be public for this peer review?**   For information about this choice, including consent withdrawal, please see our Privacy Policy

Reviewer #1: No

Reviewer #2: **Yes:**  Partha Sarathi Tripathy

**********

[NOTE: If reviewer comments were submitted as an attachment file, they will be attached to this email and accessible via the submission site. Please log into your account, locate the manuscript record, and check for the action link "View Attachments". If this link does not appear, there are no attachment files.]

To ensure your figures meet our technical requirements, please review our figure guidelines: https://journals.plos.org/plosone/s/figures

You may also use PLOS’s free figure tool, NAAS, to help you prepare publication quality figures: https://journals.plos.org/plosone/s/figures#loc-tools-for-figure-preparation.

NAAS will assess whether your figures meet our technical requirements by comparing each figure against our figure specifications.

# Author comment: Author response to Decision Letter 1

**DOI:** 10.1371/journal.pone.0355950.r002

8 Jul 2026

Response to Reviewers

Manuscript ID: PONE-D-26-16301

Revised title: Putative anaerobic transformation pathway of microcystin-RR inferred from ¹⁵N labeling and multi-omics in an enriched shrimp pond sediment microbial community

Dear Editor and Reviewers,

We sincerely thank the Academic Editor and the two reviewers for their careful reading and constructive comments. We have revised the manuscript point by point in response to all comments. In this revision, no new experiments were added; instead, we focused on (i) rigorously moderating claims that exceeded the available evidence, (ii) adding and repositioning key recent literature, (iii) rewriting the Abstract, results synthesis, Discussion, and Conclusions to match the strength of the evidence, and (iv) standardizing figure legends, table headers, molecular-formula formatting, and file presentation. All revised text is highlighted in red in the marked version of the manuscript.

Please note that the “empty sections” and “malformed molecular formulas” noted by the reviewers arose from character/layout dislocation when the submission system converted the source file into the merged PDF. The original .docx file was complete and correctly formatted; we clarify this in the relevant items below and have re-checked the corresponding sections in the revised manuscript.

For clarity, each reviewer comment is quoted below, followed by our response. Newly added references are cited using the numbering of the revised reference list.

Response to the Academic Editor

Editor Comment. Major revision is recommended. Please address all reviewer comments carefully, improve data presentation, and strengthen the discussion.

Response: We have addressed all reviewer comments point by point. Without adding new experiments, we rewrote the Abstract, results synthesis, Discussion, and Conclusions strictly within the boundaries of the available evidence; systematically moderated overstated mechanistic claims; added key recent literature; expanded the methodological rationale; and strengthened the discussion of limitations. We also standardized the figure legends, table headers, molecular-formula formatting, and the description of Fig 6 to improve the readability and internal consistency of the manuscript.

Response to Journal Requirements

1. Please ensure that your manuscript meets PLOS ONE style requirements, including those for file naming.

Response: We have checked and adjusted the manuscript against the PLOS ONE main-body and title/authors/affiliations templates. Figures, tables, and supporting information are numbered and named according to PLOS ONE conventions (Fig 1–6, Table 1, S1–S2 Table, S1–S5 Fig), and the submission files are named as required.

2. We note that the grant information provided in the Funding Information and Financial Disclosure sections do not match. Please provide the correct grant numbers in the Funding Information section.

Response: We have re-checked the funding information. The awards and grant numbers for this study are as follows, and we will update the online Funding Information section accordingly so that it fully matches the Financial Disclosure: National Natural Science Foundation of China (No. 32172978); Natural Science Foundation of Tianjin (25JCYBJC00800); Tianjin Science and Technology Planning Project (24ZYCGSN00140); Gansu Science and Technology Project (No. 24CXNP011 and 24CXNP005); and the Innovation Training Program Project for College Students of Tianjin Agricultural University (No. 202410061160).

3. Financial disclosure – funder role and author affiliation with the funder.

Response: We confirm that the funders provided financial support only and had no role in study design, data collection and analysis, decision to publish, or preparation of the manuscript. We will confirm the corresponding statement in the cover letter and will accurately complete the author-contributions information in the online form. The suggested financial-disclosure statement is: “This work was supported by the National Natural Science Foundation of China (No. 32172978), the Natural Science Foundation of Tianjin (25JCYBJC00800), the Tianjin Science and Technology Planning Project (24ZYCGSN00140), the Gansu Science and Technology Project (No. 24CXNP011 and 24CXNP005), and the Innovation Training Program Project for College Students of Tianjin Agricultural University (No. 202410061160). The funders had no role in study design, data collection and analysis, decision to publish, or preparation of the manuscript.”

4. Please amend the manuscript submission data to include authors Jingjing Zhang, Rui Chen, Wenjie Zhao, Dajuan Zhang, Qian Wang, and Xinyu Wang.

Response: The complete author list has been confirmed in the manuscript. We will update the author information in the online submission system via Edit Submission to include Jingjing Zhang, Rui Chen, Wenjie Zhao, Dajuan Zhang, Qian Wang, and Xinyu Wang, so that the system record matches the manuscript.

5. If the reviewer comments include a recommendation to cite specific previously published works, please review and evaluate these publications for relevance.

Response: We have evaluated the works suggested by the reviewers and consider them directly relevant; they have been cited accordingly: Yang et al. (2025) [37], An et al. (2026) [55], and Chen et al. (2020) [60]. The reference list has been renumbered in order of first appearance in the text, and all in-text citations correspond consistently to the final list.

Response to Reviewer #1

We thank the reviewer for recognizing the value of integrating isotope tracing with multi-omics and for the constructive concerns raised. We respond to each point below.

Comment 1. The manuscript heavily relies on correlations between metatranscriptomic upregulation and inferred enzymatic functions to propose a novel anaerobic degradation pathway. No direct biochemical or genetic validation is provided to confirm the roles of U32, M23, or S9 peptidases. The conclusions should be supported with functional evidence.

Response: We fully agree. As functional validation experiments (enzyme assays, gene knockouts, or heterologous expression) are beyond the scope of the present study, we have moderated the corresponding claims to match the available evidence and now explicitly frame them as candidate hypotheses derived from multi-omics associations rather than established mechanisms. Specifically: (i) in the Abstract, “constituting an independent anaerobic degradation pathway” was changed to “suggesting the involvement of a non-canonical, mlr-independent peptidase system in MC-RR transformation”; (ii) in the Results and Conclusions, the peptidases are consistently described as “candidate hydrolases,” with an added statement that the inferred enzymatic roles require targeted biochemical, genetic, and biogeochemical validation; and (iii) the qualifying statement that the inference lacks direct enzyme–substrate validation has been retained and emphasized in the Discussion. Heterologous expression and in vitro enzyme assays have been identified as clear next steps.

Comment 2. Only three products are tentatively assigned and many peaks remain unresolved. The proposed pathway is incomplete and lacks structural confirmation. Inferring specific cleavage sites (Arg–Adda or Ala–Arg) is not sufficiently convincing.

Response: We agree. We now clarify that product assignments are based on accurate-mass (MS1) matching within ±5 ppm and are therefore tentative, because the MS/MS fragmentation and NMR data required for unambiguous confirmation of the cleavage sites were not obtained in this study. The cleavage sites are now described as “tentatively inferred,” and the process is presented as “a multi-step transformation initiated by specific peptide-bond cleavage” rather than a fully resolved linear pathway. The three key products are labeled “tentative” in the Abstract, and the full set of 20 candidate peaks (mass, retention time, and time-series peak areas) is provided in S2 Table.

Comment 3. The claim of DNRA activation and carbon–nitrogen coupling is based solely on gene expression data, without measurement of nitrogen species or isotope tracing of nitrogen flux.

Response: We accept this criticism and have systematically moderated the relevant statements. (i) In the Abstract, the definitive statements on DNRA activation, conversion of organic nitrogen into recoverable ammonium, and efficient carbon–nitrogen coupling were replaced with “narG–nirB–nrfA and norB were concurrently upregulated, a transcriptional pattern consistent with DNRA-linked nitrogen turnover and NO detoxification during degradation, although the corresponding nitrogen fluxes were not directly measured.” (ii) In the Results, “the DNRA pathway reduced organic nitrogen to ammonium” was changed to “upregulation of DNRA-pathway genes suggested that organic nitrogen may be reduced to ammonium,” with a statement that the integrated scheme is a model requiring biogeochemical confirmation. (iii) The Discussion explicitly states that nitrate, nitrite, and ammonium dynamics were not measured and that no ¹⁵N nitrogen-flux tracing was performed; therefore, only a transcriptional response consistent with DNRA can be claimed, not confirmed DNRA flux.

Comment 4. The initial MC-RR concentration (1 microgram/mL) is not representative of most natural environments, raising concerns about ecological relevance and scalability.

Response: We have added the rationale for this concentration in the Materials and methods. Although higher than levels typically encountered in ambient surface waters, 1 µg/mL was deliberately selected to ensure sufficient accumulation of transient degradation intermediates for reliable LC-MS detection and pathway reconstruction, consistent with previous mechanistic studies of MC biodegradation [19,22]. We also note that intracellular MC released during bloom collapse can substantially elevate local concentrations at the sediment–water interface. As the present study focuses on mechanistic resolution rather than quantitative degradation kinetics, degradation efficiency at environmentally realistic concentrations will be examined in future work, and we have moderated statements regarding direct application value accordingly.

Comment 5. The role of QS under anaerobic conditions (Bioresour. Technol., 2026, 442, 133668) should be covered. The manuscript attributes a central regulatory role to multiple QS systems without experimental validation; this section is largely speculative and should be reduced or supported.

Response: We thank the reviewer for this key reference, which we have cited (An et al., Bioresour. Technol. 2026, 442, 133668 [55]). We note that the regulatory role of QS under anaerobic conditions has attracted increasing attention; for example, An et al. showed that manipulating QS through quorum quenching altered enzymatic activities and metabolic pathways in an anaerobic reactor. We have also substantially reduced the speculative framing of this section: we now state that the QS-related genes were identified solely from transcriptional co-upregulation, which indicates responsiveness but does not establish a causal regulatory role, and that QS-signal quantification, exogenous signal supplementation, or QS-inhibition/quorum-quenching assays would be required to demonstrate functional regulation. The role of QS is therefore presented as a hypothesis to be tested. The phrase “multi-layered regulatory network ensuring precise division of labor” has been removed from the Abstract.

Comment 6. The proposed syntrophic fermentation–respiration network between Citrobacter and Shewanella is inferred solely from annotation and transcriptional contribution, without co-culture, metabolite-exchange, or electron-transfer analyses.

Response: We agree and have removed the strict syntrophy designation. In the Conclusions, “establishing a syntrophic fermentation–respiration network” was changed to “the two dominant taxa exhibited complementary metabolic profiles suggestive of a cooperative fermentation–respiration relationship rather than a fully demonstrated syntrophic network.” The Discussion retains the explicit statement that only genome-annotation and transcriptional-response evidence is available and that metabolite exchange, electron transfer, and material flux were not measured; therefore, whether the relationship constitutes syntrophy in the strict sense requires confirmation by co-culture, metabolite monitoring, or isotope-flux analysis. “Precise division of labor” has been replaced by a neutral description of the low-abundance but transcriptionally active taxon.

Comment 7. Similar observations (mlr-independent degradation, diverse peptidases, community-level degradation) have been reported previously. The manuscript does not clearly delineate how its findings advance beyond prior studies.

Response: We have moderated the novelty claims (removing “novel mechanism” from the Abstract and Conclusions and replacing it with “a candidate model”) and now explicitly define the incremental contribution of this study rather than claiming a new pathway. mlr-independent and community-level degradation have indeed been reported; our incremental contribution is: (i) using ¹⁵N labeling combined with time-series LC-MS to provide product-level evidence for anaerobic MC-RR (rather than the more commonly studied MC-LR) degradation; (ii) integrating metagenomic binning with metatranscriptomics to match degradation-related candidate peptidases (U32/M23/S9 and other MEROPS families) with specific taxa and to resolve strain-level transcriptional contributions (S. chilikensis, at only 1.36% abundance, contributed approximately 28% of upregulated genes); and (iii) positioning these results against the most recent work, including Yang et al. (2025) [37]. We now state that different taxa use different functional genes but converge functionally, and we do not claim a single universal conserved pathway.

Comment 8. The manuscript frequently uses strong claims (novel mechanism, precise division of labor, efficient carbon–nitrogen coupling) that are not fully supported.

Response: We fully agree and have revised these expressions throughout: (i) “novel mechanism” to “a candidate model”; (ii) “precise division of labor” to “functional division of labor / complementary metabolic profiles,” with the phrase “ensuring precise division of labor” deleted; (iii) “efficient carbon–nitrogen metabolic coupling” to “a transcriptional pattern consistent with … / a potential coupled carbon–nitrogen linkage”; and (iv) “activated” to “concurrently upregulated.” These changes are concentrated in the Abstract, Results, and Conclusions.

Comment 9. The abstract is overly dense and contains excessive mechanistic claims not fully supported in the main text.

Response: The Abstract has been rewritten. We removed unsupported mechanistic assertions (“constituting an independent pathway,” “the DNRA pathway was activated … achieving efficient carbon–nitrogen coupling,” and “a multi-layered regulatory network ensuring precise division of labor”), added “tentative” to the product assignments, and revised the descriptions of the peptidases, DNRA, and QS to evidence-matched wording (“suggesting/consistent with/concurrently upregulated”), while adding the key qualifier that nitrogen fluxes were not directly measured. The revised Abstract retains the core findings while markedly reducing the density and strength of the claims.

Comment 10. Figures (especially pathway diagrams) are conceptual and lack quantitative support.

Response: We agree that Fig 6 is essentially an integrative conceptual model. To avoid misinterpretation, we added a clarifying statement to the Fig 6 legend: the scheme is a conceptual model integrating LC-MS product identification with metagenomic and metatranscriptomic evidence, and the transcription-based steps are inferential and require further functional validation. We note that the quantitative differential-expression information underlying the model is provided by Fig 5 (heatmap of DEGs for peptidases, carbon/nitrogen metabolism, amino-acid biosynthesis, and QS) and Fig 4 (volcano plot); Fig 6 is used to present the metabolic logic suggested by these quantitative results and is referred to as a

Attachment Submitted filename: Response_to_Reviewers_1.docx

# Peer review: Decision Letter 1

**DOI:** 10.1371/journal.pone.0355950.r003

**Authors:** Bijay Kumar Behera

28 Jul 2026

Putative anaerobic transformation pathway of microcystin-RR inferred from ¹⁵N labeling and multi-omics in an enriched shrimp pond sediment microbial community

PONE-D-26-16301R1

Dear Dr. Liu,

We’re pleased to inform you that your manuscript has been judged scientifically suitable for publication and will be formally accepted for publication once it meets all outstanding technical requirements.

Within one week, you’ll receive an e-mail detailing the required amendments. When these have been addressed, you’ll receive a formal acceptance letter and your manuscript will be scheduled for publication.

An invoice will be generated when your article is formally accepted. Please note, if your institution has a publishing partnership with PLOS and your article meets the relevant criteria, all or part of your publication costs will be covered. Please make sure your user information is up-to-date by logging into Editorial Manager at Editorial Manager®  and clicking the ‘Update My Information' link at the top of the page. For questions related to billing, please contact billing support .

If your institution or institutions have a press office, please notify them about your upcoming paper to help maximize its impact. If they’ll be preparing press materials, please inform our press team as soon as possible -- no later than 48 hours after receiving the formal acceptance. Your manuscript will remain under strict press embargo until 2 pm Eastern Time on the date of publication. For more information, please contact onepress@plos.org.

Kind regards,

Bijay Kumar Behera, Ph.D.

Academic Editor

PLOS One

Additional Editor Comments (optional):

Dear Dr. Liu,

Thank you for submitting the revised version of your manuscript entitled, "Putative anaerobic transformation pathway of microcystin-RR inferred from ¹⁵N labeling and multi-omics in an enriched shrimp pond sediment microbial community" to PLOS ONE.

The revised manuscript has now been evaluated in light of the reviewers' comments and your detailed responses. I appreciate the considerable effort you and your co-authors have made in addressing the concerns raised during the initial review. The revisions have substantially improved the clarity, scientific rigor, and overall quality of the manuscript.

Both reviewers have evaluated the revised version positively and recommend acceptance. The responses to the reviewers are comprehensive, and the revisions satisfactorily address the major and minor issues identified during the previous review round. The study presents a valuable contribution by elucidating the putative anaerobic transformation pathway of microcystin-RR through the integration of stable isotope labeling (¹⁵N) and multi-omics approaches in an enriched shrimp pond sediment microbial community. The findings enhance our understanding of anaerobic cyanotoxin biodegradation and provide important insights with potential implications for environmental remediation and aquaculture ecosystem management.

Based on the positive reviewer recommendations and my own assessment of the revised manuscript, I am pleased to inform you that your manuscript is accepted for publication in PLOS ONE.

Congratulations to you and your co-authors on this excellent work. Thank you for choosing PLOS ONE to publish your research, and I look forward to seeing your article published.

Sincerely,

Bijay Kumar Behera, Ph.D.

Academic Editor

PLOS ONE

Reviewers' comments:

Reviewer's Responses to Questions

**Comments to the Author**

Reviewer #1: (No Response)

Reviewer #2: All comments have been addressed

**********

2. Is the manuscript technically sound, and do the data support the conclusions?

Reviewer #1: (No Response)

Reviewer #2: Yes

**********

3. Has the statistical analysis been performed appropriately and rigorously?

Reviewer #1: (No Response)

Reviewer #2: Yes

**********

4. Have the authors made all data underlying the findings in their manuscript fully available?

The PLOS Data policy

Reviewer #1: (No Response)

Reviewer #2: Yes

**********

5. Is the manuscript presented in an intelligible fashion and written in standard English?

Reviewer #1: (No Response)

Reviewer #2: Yes

**********

Reviewer #1: The comments raised by the reviewers have been addressed, thus can be accepted for possible publication.

Reviewer #2: All the comments have been addressed properly by the authors and the manuscript can be accepted in the present form

**********

what does this mean? ). If published, this will include your full peer review and any attached files.

If you choose “no”, your identity will remain anonymous but your review may still be made public.

**Do you want your identity to be public for this peer review?**   For information about this choice, including consent withdrawal, please see our Privacy Policy

Reviewer #1: No

Reviewer #2: **Yes:**  Partha Sarathi Tripathy

**********

# Editor report: Acceptance letter

**DOI:** 10.1371/journal.pone.0355950.r004

**Authors:** Bijay Kumar Behera

PONE-D-26-16301R1

PLOS One

Dear Dr. Liu,

I'm pleased to inform you that your manuscript has been deemed suitable for publication in PLOS One. Congratulations! Your manuscript is now being handed over to our production team.

At this stage, our production department will prepare your paper for publication. This includes ensuring the following:

* All references, tables, and figures are properly cited

* All relevant supporting information is included in the manuscript submission,

* There are no issues that prevent the paper from being properly typeset

You will receive further instructions from the production team, including instructions on how to review your proof when it is ready. Please keep in mind that we are working through a large volume of accepted articles, so please give us a few days to review your paper and let you know the next and final steps.

Lastly, if your institution or institutions have a press office, please let them know about your upcoming paper now to help maximize its impact. If they'll be preparing press materials, please inform our press team within the next 48 hours. Your manuscript will remain under strict press embargo until 2 pm Eastern Time on the date of publication. For more information, please contact onepress@plos.org.

You will receive an invoice from PLOS for your publication fee after your manuscript has reached the completed accept phase. If you receive an email requesting payment before acceptance or for any other service, this may be a phishing scheme. Learn how to identify phishing emails and protect your accounts at https://explore.plos.org/phishing.

If we can help with anything else, please email us at customercare@plos.org.

Thank you for submitting your work to PLOS ONE and supporting open access.

Kind regards,

PLOS ONE Editorial Office Staff

on behalf of

Dr. Bijay Kumar Behera

Academic Editor

PLOS One
